# Indole-Based Compounds in the Development of Anti-Neurodegenerative Agents

**DOI:** 10.3390/molecules29092127

**Published:** 2024-05-03

**Authors:** Elisabetta Barresi, Emma Baglini, Valeria Poggetti, Jacopo Castagnoli, Doralice Giorgini, Silvia Salerno, Sabrina Taliani, Federico Da Settimo

**Affiliations:** 1 Department of Pharmacy, University of Pisa, Via Bonanno 6, 56126 Pisa, Italy; elisabetta.barresi@unipi.it (E.B.); valeria.poggetti@phd.unipi.it (V.P.); jacopo.castagnoli@phd.unipi.it (J.C.); federico.dasettimo@unipi.it (F.D.S.); 2 Institute of Clinical Physiology, National Research Council of Italy, CNR Research Area, 56124 Pisa, Italy; emma.baglini@cnr.it; 3 Department of Pharmacy, University of Salerno, Via Giovanni Paolo II 132, Fisciano, 84084 Salerno, Italy; dgiorgini@unisa.it

**Keywords:** neurodegeneration, indole nucleus, multifunctional compounds

## Abstract

Neurodegeneration is a gradual decay process leading to the depletion of neurons in both the central and peripheral nervous systems, ultimately resulting in cognitive dysfunctions and the deterioration of brain functions, alongside a decline in motor skills and behavioral capabilities. Neurodegenerative disorders (NDs) impose a substantial socio-economic strain on society, aggravated by the advancing age of the world population and the absence of effective remedies, predicting a negative future. In this context, the urgency of discovering viable therapies is critical and, despite significant efforts by medicinal chemists in developing potential drug candidates and exploring various small molecules as therapeutics, regrettably, a truly effective treatment is yet to be found. Nitrogen heterocyclic compounds, and particularly those containing the indole nucleus, which has emerged as privileged scaffold, have attracted particular attention for a variety of pharmacological applications. This review analyzes the rational design strategy adopted by different research groups for the development of anti-neurodegenerative indole-based compounds which have the potential to modulate various molecular targets involved in NDs, with reference to the most recent advances between 2018 and 2023.

## 1. Introduction

Neurodegenerative diseases (NDs) comprise increasingly widespread diverse neurological disorders: millions of people around the world are affected by these diseases that cause severe dysfunctions especially in older people [1]. The increase in NDs is mainly due to the growth and aging of the global population. If the cases continue to increase, by 2025 more than 65% of people over the age of 65 will be affected by these diseases [2]. Despite a decline in communicable NDs, over the past 30 years, the number of deaths has increased by 39%. As predicted by the World Health Organization (WHO), NDs will overtake cancer within 20 years and become the second leading cause of death, after cardiovascular disease. Therefore, the need to find an effective therapeutic approach for NDs is quite urgent because, to date, commercially available drugs for NDs provide only transient relief from symptoms and they do not offer a cure or a halt to the progression of the disease [3,4].

Neurodegeneration is a process that causes a continuous loss of structure, function and quantity of neurons in the central and peripheral nervous system, leading to cognitive impairment and deterioration of the brain, as well as motor and behavioral decline [4]. 

The most common NDs are Alzheimer’s disease (AD) and Parkinson’s disease (PD), without forgetting Huntington’s disease (HD), amyotrophic lateral sclerosis (ALS), multiple sclerosis (MS) and other psychological disorders. Oxidative stress, mitochondrial dysfunction, neuronal loss, viral inflammation, ischemia-reperfusion injury and anomalous protein aggregation in the neuronal tissues may be the common causes of all these diseases [4]. 

Specifically, AD is characterized by the presence of senile plaques (SPs) and neurofibrillary tangles (NFTs), consisting of amyloid-β (Aβ) and hyperphosphorylated tau (p-tau) aggregates, respectively. PD is associated with the accumulation of misfolded aggregates of α-synuclein (α-Syn) in Lewy bodies. ALS exhibits aggregates of mutant superoxide dismutase 1 (SOD1), TAR DNA binding protein 43 (TDP-43), fused in sarcoma (FUS), and repeated dipeptides resulting from the non-canonical translation of mutant chromosome 9 open reading frame 72 (C9ORF72). Additionally, metal ion dyshomeostasis in the brain of individuals experiencing neurodegeneration is a noteworthy aspect [5].

The underlying causes of NDs are complex and multifactorial, involving a combination of genetic, environmental, and lifestyle factors. While there are treatments available to manage symptoms and slow disease progression for some NDs, there is currently no cure for most of these conditions. Ongoing research aims to better understand the mechanisms underlying these diseases and develop more effective treatments.

Until now, numerous years of extensive research have resulted in the discovery of genetic factors and shared biochemical pathways in various NDs. These findings have unveiled eight distinctive hallmarks associated with NDs: pathological protein aggregation, dysfunction in synaptic and neuronal networks, aberrant proteostasis, cytoskeleton abnormalities, altered energy homeostasis, DNA and RNA defects, inflammation, and neuronal cell death [6]. 

In many NDs the characteristic protein aggregation is found in brain regions correlating with clinical outcomes. Thus, protein aggregation is a hallmark of NDs, resulting also useful for the diagnosis and classification of the disease.

Symptoms of NDs typically reflect disturbance of specific neuronal networks; in fact, synaptic insufficiency appear to be an early event in many NDs.

In NDs the accumulation of aggregated proteins further implicates aberrant proteostasis, which is regulated by two main cellular mechanisms: the ubiquitin-proteasome system (UPS) and the autophagy-lysosome pathway (ALP). These mechanisms are closely intertwined with cell death pathways and have been identified as responsible for neuronal death. Since neurons are highly active and consequently have high energy demands, altered energy homeostasis is also involved in several NDs. In addition, neuronal cytoskeletal abnormalities, and related functions, such as axonal transport, play a central role in NDs. 

Several neurological diseases are due to defects in the ability to eliminate genomic stress and this confirms the involvement of DNA damage in neurodegeneration. Moreover, based on experimental proof, an important role for RNA dysregulation in several NDs has been evidenced. 

Many data indicate a well-characterized neuroinflammatory component in NDs [7], so classifying inflammation as an ND hallmark and also highlighting its close interconnection with other ND’s hallmarks.

**Figure 1:**
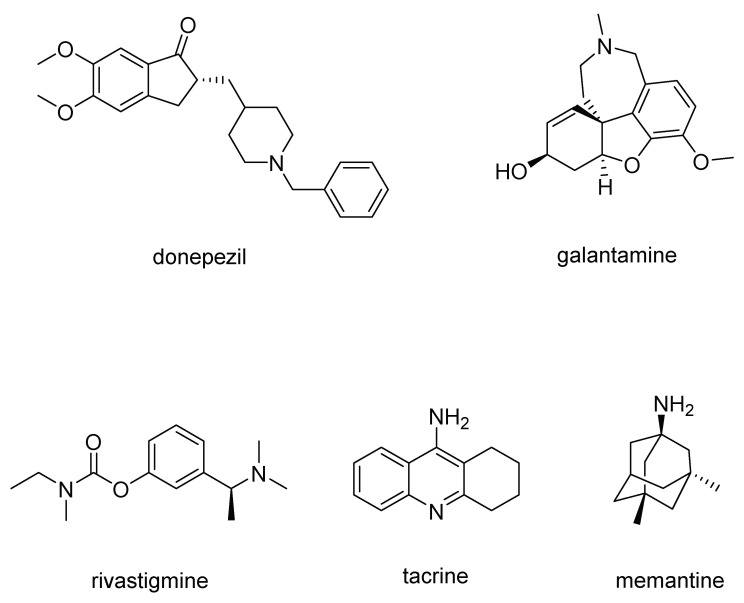
Chemical structures of drugs used for AD treatment.

In NDs some intrinsic properties of neurons may make them particularly vulnerable to cell death; moreover, all the ND’ hallmarks may contribute to neuronal cell loss, giving rise to some pathological and clinical manifestations [6]. Currently, the approved medications for AD include acetylcholinesterase (AChE) inhibitors, an NMDA receptor antagonist, and two monoclonal antibodies targeting amyloid deposition. Among these, there are three orally administered drugs: donepezil and galantamine (Figure 1), both of which are rapid-acting reversible AChE inhibitors, and rivastigmine (Figure 1), a slow-acting reversible inhibitor of both AChE and butyrylcholinesterase (BChE) [3].

Tacrine (Figure 1), an AChE inhibitor was also used against AD, but due to its hepatotoxic side effects, currently, it is no longer used in clinical practice [8]. 

Memantine (Figure 1) is an NMDA receptor antagonist which avoids the pathological increase in glutamate and slows the accumulation of intracellular calcium. It is administered orally, also in association with AChE inhibitors, resulting in improved patient status, with synergistic effects without increased adverse effects.

Aducanumab, an IgG1 monoclonal antibody, represents a significant milestone, being the first drug designed to target one of the underlying causes of AD, that is Aβ soluble oligomers and insoluble fibrils [9,10]. This approach leads to a reduction in amyloid plaques and a decrease in phosphorylated tau levels in the cerebrospinal fluid [11]. However, the clinical effectiveness and safety of aducanumab in AD have been a subject of debate. While the FDA has granted approval to the drug, classifying it as a top-tier medication, the EMA has rejected its approval [12]. 

In a similar vein, lecanemab, another humanized IgG1 monoclonal antibody with high-affinity binding to Aβ soluble protofibrils [13], has demonstrated the ability to mitigate mild cognitive decline and reduce Aβ plaques in patients with early AD.

**Figure 2:**
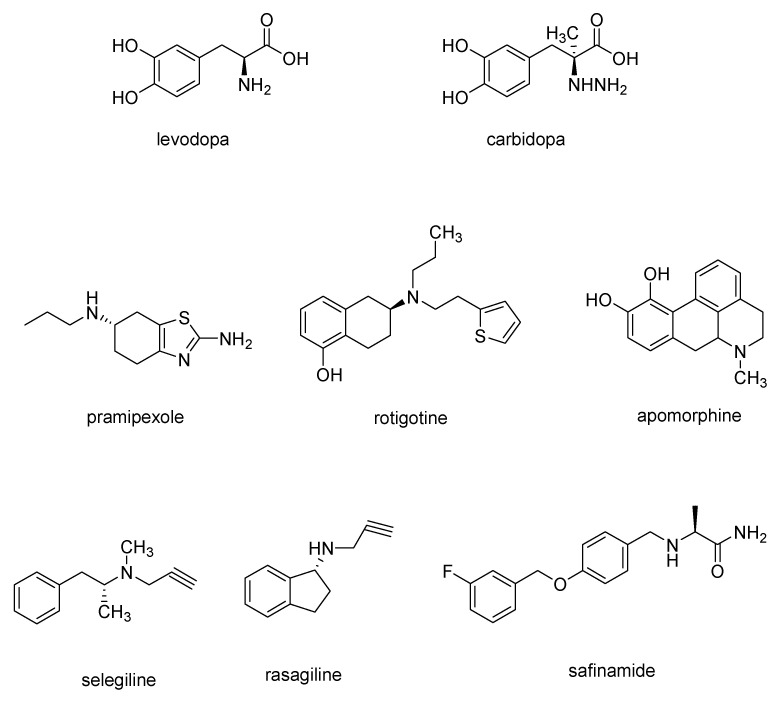
Chemical structures of drugs used for PD treatment.

Since the loss of the number of dopaminergic neural cells in the substantia nigra is found to be one of the main cause of PD symptoms [14], the most widely used drugs for PD treatment are levodopa-based drugs which are able to cross the blood–brain barrier (BBB) and, then, in the brain, are converted to dopamine. Often, levodopa (Figure 2) is combined with carbidopa (Figure 2), a DOPA decarboxylase inhibitor, to avoid levodopa decarboxylation to dopamine before it can permeate BBB in order to increase the amount of levodopa in the central nervous system (CNS) [15]. 

Dopamine receptor agonists, which replicate the effects of dopamine in the brain, may not be as potent as levodopa in symptom management. Nevertheless, they have a more extended duration of action and can be utilized in conjunction with levodopa to alleviate the occasional on-off fluctuations associated with levodopa treatment. This category encompasses pramipexole, rotigotine, and apomorphine (Figure 2), a rapidly acting dopamine agonist administered via injection for rapid relief [15]. 

Finally, monoamine oxidase B (MAO-B) inhibitors, such as selegiline, rasagiline and safinamide (Figure 2) aims to alleviate PD symptoms by inhibiting the brain enzyme MAO-B, so preventing the breakdown of dopamine in the brain.

Although medicinal chemists have dedicated huge efforts in developing drug candidates for NDs and diverse small molecules have been explored as potential therapeutics, unfortunately, actually, an effective drug has not been identified yet [4,16]. 

As widely documented, heterocyclic compounds serve as the basic building blocks to obtain molecules endowed with suitable pharmacotherapeutic properties [17,18,19,20,21,22,23]. In particular, nitrogen-containing heterocycles, as recently highlighted by the FDA, resulted the most interesting in drug design since they can be considered both acceptors and donors of hydrogen bonds, thus effectively binding various molecular targets and exerting important pharmacological effects [20,21]. 

In this context, indole is a naturally occurring heterocyclic aromatic ring system found in both plants and animals. It consists of a six-membered benzene ring fused with a five-membered pyrrole ring, possessing electronic and steric characteristics which make it favorable for achieving bioavailability and pharmacological effects. Indole is recognized as a versatile pharmacophore due to its ability to interact with various receptors, giving rise to a wide range of biological activities. As a result, it is considered one of the most privileged frameworks in the field of medicinal chemistry [24]. 

In the development of more modern chemotherapy drugs, medicinal chemists were attracted by the clinical success and adaptability of the indole nucleus and in fact this nucleus is present in many therapeutic agents endowed with anti-HIV, anti-inflammatory, antihypertensive, antileukemia, anti-psychotic, antiviral, antimigraine, antiemetic, anti-asthmatic and anti-neurodegenerative activity [19,25,26].

The purpose of this review would be to update previous papers [25,27], reporting indole-based compounds as anti-neurodegenerative agents.

In the next section, we will focus on the rational design strategies employed by various research teams in the development of anti-neurodegenerative indole-based compounds which have the potential to modulate various molecular targets, with reference to the most recent advances between 2018 and 2023.

## 2. Indole-Based Compounds as Potential Anti-Neurodegenerative Agents

Although enormous efforts have been made to develop drug candidates for treating NDs, an effective therapeutic has not been identified yet. Anyhow, diverse small molecules have been explored as potential therapeutics mainly for AD and PD, the two top NDs.

In this section, an overview of recently (2018–2023) reported indole-containing molecules that have been evaluated as potential candidates for the treatment of NDs was provided. When possible, structure–activity relationships (SARs) are also discussed.

All the indole compounds herein reported have been categorized into different classes based on the corresponding target protein; finally, the multifunctional indole derivatives have been also reported, i.e., single molecules or hybrid molecules acting as multitarget-directed ligands (MTDLs).

### 2.1. Cholinesterase (ChE) Inhibitors

The cerebral cortex of AD brains exhibits a depletion of acetylcholine (ACh), leading to the degeneration of cholinergic neurons and impaired cholinergic neurotransmission [28]. AChE, which hydrolyzes ACh into acetate and choline within neurons [29], is predominantly found at the synaptic cleft and neuromuscular junction in the brain, while BChE, which hydrolyzes various choline or non-choline esters and amides in a nonspecific manner [30], is primarily present in blood plasma outside the CNS. Since the coexistence of AChE or BChE with Aβ aggregates in senile plaques (SPs) can accelerate Aβ aggregation, thereby exacerbating neurotoxicity [31,32,33], ChE inhibitors have emerged as promising targets for AD treatment, leading to the development of FDA-approved drugs for AD treatment, such as donepezil (Aricept), rivastigmine (Exelon), and galantamine (Reminyl) (Figure 1) [27]. 

**Figure 3:**
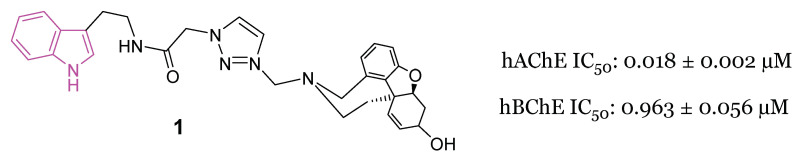
Chemical structure and biological activity of indole-based dual inhibitor of ChEs **1**.

In 2021, Molęda et al. [34] reported the development of novel dual-binding inhibitors of ChE, designed to selectively target the catalytic and peripheral anionic sites (CAS and PAS) of AChE. Among the 12 hybrids synthesized and evaluated for their efficacy against both human enzymes, hAChE and hBChE, compound **1** (Figure 3) emerged as the most potent ChE inhibitor, exhibiting an IC_50_ value of 0.018 µM for hAChE and 0.963 µM for hBChE.

**Figure 4:**
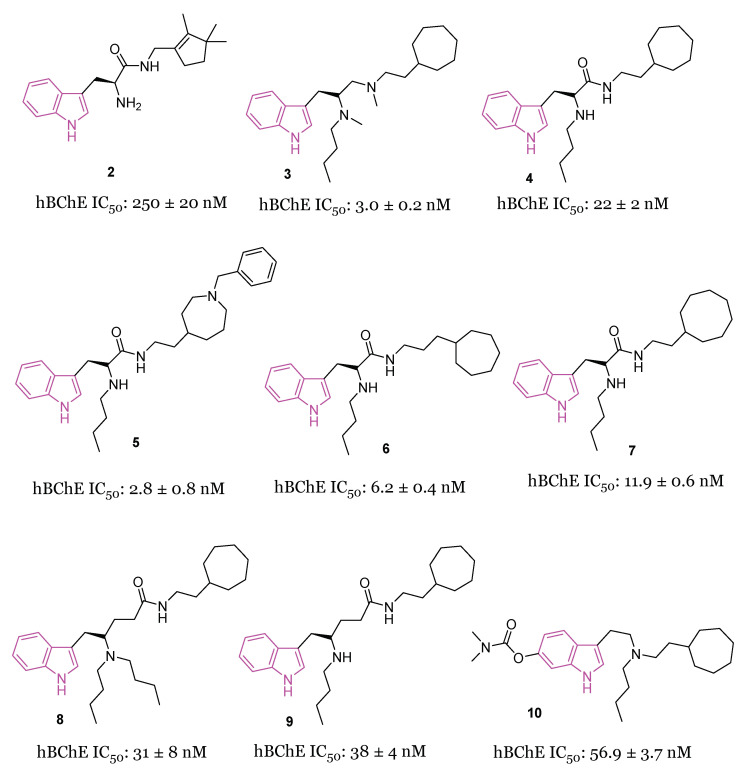
Chemical structures and biological activities of tryptophan-based selective BChE inhibitors **2**–**10**.

A large study was carried out starting from 2016, following the discovery of tryptophanamide **2** (Figure 4) as a potent inhibitor of BChE in an initial screening of a library derived from (+)-isocampholenic acid, in which this compound served as lead for the development of a virtual combinatorial library comprising 399 analogs [35]. Further investigations into the potential of tryptophan-derived compounds as BChE inhibitors were elaborated upon in a subsequent study by A. Meden et al. [36]. To explore the diverse chemical landscape presented by the initial hit **2** (Figure 4) the focus shifted towards indole-derived amides. Various modifications in the linker length between the ring systems, indole substitution patterns, and analogues of the isocampholenic acid-derived ring were investigated. These modifications generally resulted in decreased activity against BChE compared to the hit **2**, while selectivity over AChE was maintained. However, upon N-alkyl derivatization of the α-amino group, there was a notable increase in activity against BChE, with consistent selectivity over AChE. Further enhancements were observed with additional alkyl substituents, albeit with reduced potency upon removal of the α-amino basic center. Elongation of the alkyl linker and enlarging the cycloalkyl ring yielded potent inhibitors, with the tertiary diamine **3** (Figure 4) emerging as the most potent of the series. Investigation on the central indole core revealed that certain modifications, such as oxidation or reduction, led to diminished BChE inhibition. Similarly, substitutions with indazole, quinoline, and 4-phenyltriazole resulted in reduced potency. Conversely, the β-naphthylalanine derivative exhibited greater BChE inhibition compared to its tryptophan analogue **4** (Figure 4). The crystal structure of compound **3** in complex with human BChE revealed a novel binding mode, distinct from those previously reported [37]. 

In initial assessments of cytotoxicity using the SH-SY5Y neuroblastoma cell line, compound **3** exhibited minimal cytotoxic effects, displaying LD_50_ values within the micromolar spectrum. Additionally, it demonstrated high permeability across the BBB, as anticipated through the in vitro PAMPA-BBB assay [36]. 

Recently [38], a subsequent investigation into a novel series of tryptophan-based selective hBChE inhibitors was conducted to expand upon the previously established SARs. This study aimed to optimize compounds for potency, selectivity, and synthetic accessibility, referring to compound **4** [36] as the lead compound. The introduction of endo- or exocyclic tertiary amine at the end of the seven-membered ring did not lead to improved inhibition. As was later evident from the resolved crystal complexes, this was due to the fact that the ring positioned itself in the enzyme pocket in a hydrophobic region in such a way that the cationic interaction with Trp82 could not be effected [36]. It has been hypothesized that a flexible side chain with cation or π donor at the far end of the ring could reach Trp82 and, among the series, compound **5** (Figure 4) raised as a potent selective inhibitor of BChE (IC_50_ = 2.8 nM) [38]. The crystal structure analysis of inhibitor **5** complexed with BChE elucidated the molecular basis for its potent low nanomolar inhibition. Through systematic exploration of chemical space and guided by structure-based design using X-ray crystallography, this series was refined to enhance inhibitory potency. Structural modifications unveiled in this research delineated the critical recognition and selectivity determinants, as well as the constraints of the scaffold employed. The pronounced selectivity of the tryptophan-derived compounds for hBChE over hAChE was attributed to the indole moiety, which occupies the hydrophobic acyl-binding pocket and engages in π-π interactions with Phe329 and Trp231. To verify the significance of this interaction, compounds **6** and **7** (Figure 4) were synthesized and it was found that elongation of the alkyl linker between the amide group and the aliphatic ring (**6**, IC_50_ = 6.2 nM), as well as the enlargement of the cycloalkyl ring (**7**, IC_50_ = 11.9 nM), produced potent inhibitors [36]. Meanwhile, substituting tryptophan’s indole ring with leucine’s isopropyl group (structures not reported) significantly diminished inhibitory potency by three orders of magnitude, while substitution with the smaller phenyl ring also reduced potency, albeit to a lesser extent [38]. 

Comparative analysis with the parent compound **4** showed that elongated derivatives, such as γ-aminoamide derivatives **8** and **9** (Figure 4), exhibited slightly decreased inhibition of hBChE.

Predictions based on favorable physicochemical properties and in vitro experiments suggested high BBB permeability, enabling the tryptophan-based hBChE inhibitors to reach their target in the brain. Consequently, an initial animal trial was conducted to evaluate the potential of lead compound **4** in mitigating learning and memory deficits induced by cholinergic deficits in an AD mouse model. Behavioral assays demonstrated significant improvement in fear-motivated long-term memory and spatial long-term memory retrieval mediated by **4**. Additionally, a preliminary in vivo safety pharmacology study revealed positive impacts on memory retrieval without any adverse motor effects.

In 2022, the same authors [39] studied a novel set of tryptophan-based analogs synthesizing 18 compounds and evaluating their inhibitory effects on both AChE and BChE. Among these, compound **10** (Figure 4) demonstrated the highest potency as a BChE inhibitor, with an IC_50_ value of 56.9 nM. Molecular docking studies indicated that compound **10** forms hydrogen bonds with Leu286, and its indole fragment interacts with Trp82. 

**Figure 5:**
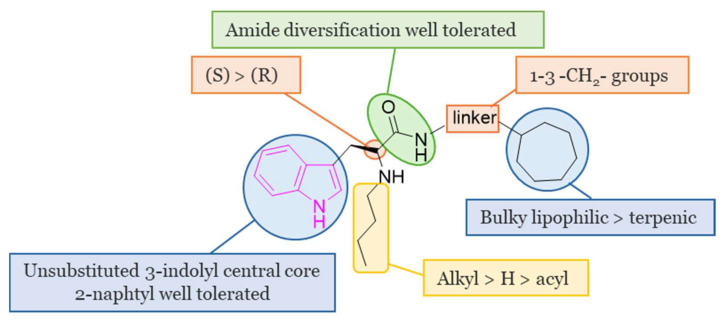
SARs of tryptophan-based selective BChE inhibitors.

The main SARs of tryptophan-based analogs as selective BChE inhibitors are depicted in Figure 5.

**Figure 6:**
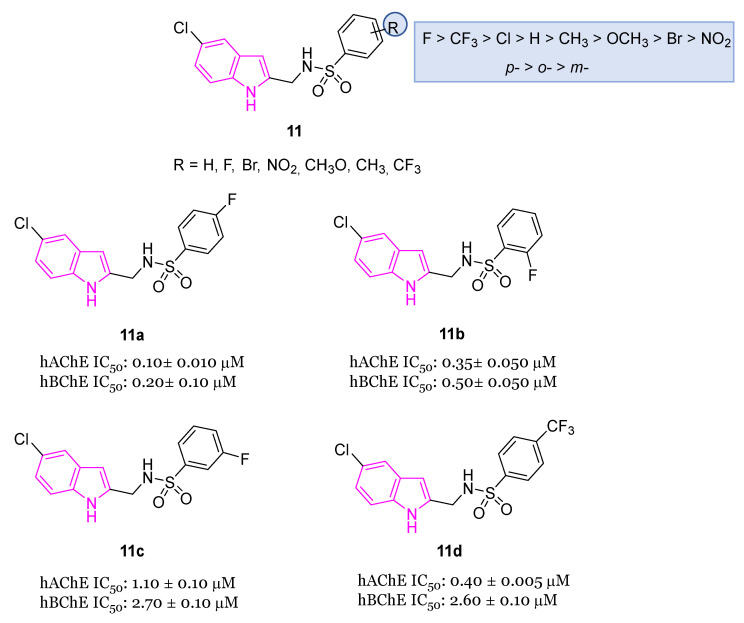
Chemical structures of indole-based sulfonamide derivatives **11, 11a**–**d** and their biological activity and SARs.

Drugs containing sulfonamides, useful in the treatment of AD, also called sulfa drugs, [40] showed excellent BChE inhibitory activity and were synthesized from previous marketed drugs, displaying an excellent biological profile at very low concentrations [37,41]. In this context, very recently [42], the synthesis and the biological activity of indole-based sulfonamide derivatives of the general formula **11** (Figure 6) were reported. All compounds with different substitution pattern (R) on the aromatic ring were evaluated for their AChE and BChE inhibition activity, showing varied degrees of inhibition profiles. In particular, their IC_50_ values were in the range 0.15–32.10 μM against AChE and 0.20–37.30 μM against BChE. Fluorinated compounds **11a**–**d** (Figure 6) resulted as the best performing compounds, with being compound **11a** the most potent one with IC_50_ values of 0.10 μM and 0.20 μM for AchE and BChE, respectively. Docking studies for the best-performing compounds **11a**–**d** against AChE evidenced different binding modalities, which could be attributable to variations in both the R substituents and their positions (o-, m- and p-). Potent inhibition of selected compounds may be due to the magnitude of activation of fluorine group (activated group): via a resonance effect, fluorine may donate some of its electron density into a conjugated π-system thus making the π-system more nucleophilic. Consequently, these electronic effects increase the electrophilic substitution reaction on aromatic ring, thus facilitating numerous interactions with key residues of AChE. However, also compound **11d** bearing deactivating group CF_3_ which removes some of the electron density from the π-system, thus making it less reactive, showed a good inhibitory activity against AChE [42]. 

**Figure 7:**
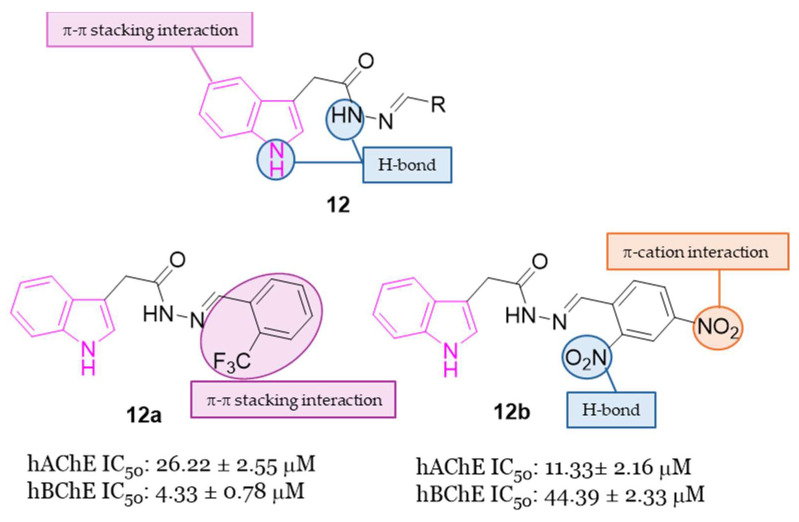
General chemical structure of indole-based hydrazide-hydrazone derivatives **12**, chemical structures of derivatives **12a**,**b** and their main interaction in the binding sites of ChEs.

Hydrazide-hydrazone scaffolds are biologically important pharmacophore groups and many compounds incorporating these moieties have been reported in the literature to show a wide range of biological activities including anticholinesterase activity [43,44,45]. In addition to the hydrophobic interactions of the indole system due to the presence of aromatic rings within the anionic sites of ChEs, the hydrazide-hydrazone linker would be a valuable moiety to form hydrogen bonds with the amino acids on the esterase or acyl binding sites. Moreover, the addition of diverse aromatic residues with a hydrazide-hydrazone bridge could be meaningful for the formation of further hydrophobic and hydrogen bond interactions with the target enzymes [43,44,45]. In this context, in 2022 the synthesis and the evaluation of AChE and BChE inhibitory activity of novel indole-based hydrazide-hydrazone derivatives **12** (Figure 7) were reported [46]. The inhibitory activity of the novel compounds was carried out on electrophorus electricus AChE (eeAChE) and equine BChE (eqBChE) enzymes, with compounds **12a** and **12b** displaying the highest AChE inhibitory activity with IC_50_ values of 26.22 μM and 11.33 μM, respectively. Furthermore, compound **12a** resulted the best BChE inhibitor of the series recording an IC_50_ value of 4.33 μM. Docking studies targeting AChE and BChE were carried out on the best performing compounds, namely derivatives **12a**,**b**, to understand their interactions with the enzymes. These studies evinced that the NH of indole ring and the NH of the hydrazone moiety formed strong hydrogen bonding interactions in the active site of ChEs, while the indole and the 2-CF_3_Ph moiety of compound **12a** formed π-π stacking interactions with two amino acid residues. In compound **12b** also the 2-NO_2_ moiety formed hydrogen bonding interactions, while the 4-NO_2_ moiety formed a π-cation interaction in the active site of the enzymes. In silico studies revealed that the pharmacokinetic properties of all the target compounds of series **12** were in accordance with the drug-likeness criteria [46]. 

### 2.2. Protein Aggregation Inhibitors

The proteopathy associated with AD is outlined through two well-established hypotheses: the amyloid cascade hypothesis and the tau hypothesis, which seem to be connected to each other. In fact, tau and phosphorylated tau (p-tau) have been shown to interact with Aβ, both directly and indirectly, and it has been proposed that NFT formation may be attributed to Aβ. The similarity in toxicity of oligomeric forms between Aβ and tau has led to the idea that these oligomeric species might collaboratively contribute to inducing neurotoxicity and synaptic dysfunction. Additionally, disturbances in calcium balance, glycogen synthase kinase 3 (GSK3), cyclin-dependent kinase-5 (cdk5), and free fatty acids have been identified as connecting factors between Aβ and tau. The interplay between Aβ and tau appears intricate, and a more thorough understanding could be gained through substances capable of interacting with both [5]. 

#### 2.2.1. The Amyloid Cascade Hypothesis

Numerous studies have suggested that Aβ, a peptide consisting of 39 to 43 amino acids and a major component of amyloid plaques, plays a crucial role in the pathophysiology of AD. Aβ is generated through the proteolysis of amyloid precursor protein (APP), a transmembrane integral glycoprotein in the CNS, following the amyloidogenic pathway. Oligomeric Aβ peptides interact with neurons and glial cells, triggering proinflammatory cascades, such as mitochondrial dysfunction, and increased oxidative stress, as well as impairing intracellular signaling and calcium regulation. Consequently, this leads to neuronal apoptosis and cell death. Therefore, one potential therapeutic strategy for AD is to prevent neuronal damage by inhibiting the aggregation of Aβ into toxic forms [27].

#### 2.2.2. The Tau Hypotheses

NFTs, another significant histopathological feature of AD, primarily consist of tau protein aggregates within neurons. The tau hypothesis associates AD toxicity with tau’s pathogenicity, as NFT burden correlates more accurately with neurodegeneration than Aβ plaque load. Tau, a phosphoprotein belonging to the microtubule-associated proteins (MAPs) family, plays a crucial role in stabilizing microtubules, supporting axonal transport, and maintaining dendrite structure. Hyperphosphorylation of tau, resulting in loss-of-function, disrupts its binding to microtubules, leading to axonal trafficking and dendrite structure deterioration. Tau aggregation progresses from oligomers and amorphous tangles to paired helical filaments (PHFs) and straight filaments (SFs), similar to Aβ fibrils. Initiation of tau aggregation has been linked to cdk5 in Tg mouse models. In this context, regulating the abnormal hyperphosphorylation and aggregation of tau has the potential to be an approach for drug discovery [5]. Beyond AD, conditions collectively referred to tauopathies (such as sporadic corticobasal degeneration, progressive supranuclear palsy, and frontotemporal dementia) have been associated with tau, indicating a shared link among various NDs [5]. 

Aducanumab, a human IgG1 monoclonal antibody, specifically designed to counter both soluble and insoluble aggregated forms of amyloid-β, was developed by Biogen for the treatment of AD. Aducanumab demonstrates efficacy in reducing brain Aβ levels in patients with AD and its effects are dependent on dosage and duration of treatment [47]. 

**Figure 8:**
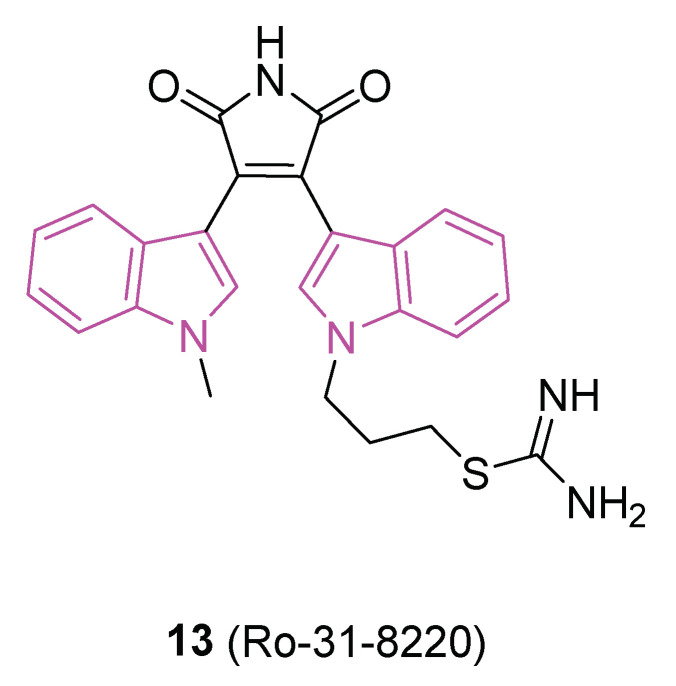
Chemical structure of protein aggregation inhibitor Ro-31-8220 (**13**).

In this context, in 2019 [48] the ability of a small custom library of therapeutics to improve a protein tau-induced rough-eye phenotype in a model of frontotemporal dementia was investigated. The tau phosphorylation in vivo was also assessed and, among the potential hits, the indole derivative **13** (Ro 31-8220, Figure 8), described earlier as a potent protein kinase C alpha (PKCα) inhibitor, was investigated. **13** greatly improved the rough eye phenotype, reduced phosphorylated tau species in vitro and in vivo, reversed tau-induced memory impairment, and improved midge motor functions. In addition, in a human neuroblastoma cell line, **13** reduced PKC activity and the tau phosphorylation pattern. Hence, **13** can be considered a novel therapeutic mitigator of tau-induced neurotoxocity.

### 2.3. Monoamine Oxidases B (MAO-B) Inhibitors 

MAOs represent enzymes located on the outer membrane of mitochondria (OMM), comprising two distinct isoforms, namely MAO-A and MAO-B, which play a crucial role in the breakdown of neurotransmitters like dopamine, serotonin, adrenaline, and noradrenaline. Clinically, inhibitors targeting MAO-B, such as selegiline and rasagiline (Figure 2), have been employed for the treatment of PD and showed promise as potential therapeutics for AD. A study conducted post-mortem on the brains of individuals with AD revealed a significant increase in MAO-B activity in specific cortical regions (frontal, parietal, and occipital cortices), thalamus, and white matter [49,50]. Elevated MAO-B activity is also associated with gliosis, leading to heightened levels of reactive oxygen species (ROS). This, in turn, promotes the production of Aβ by reducing the activity of α-secretase while simultaneously enhancing the activities of β- and γ-secretases [51]. Park and colleagues demonstrated that the persistent inhibition of MAO through selegiline, which is an irreversible and selective MAO-B inhibitor of the propargylamine type, may not lead to the enhancement of cognitive deficits in animal models of AD during prolonged treatment [52]. Hence, there is a suggestion that reversible inhibition could offer potential advantages in the development of future therapies for AD and potentially for other diseases as well.

**Figure 9:**
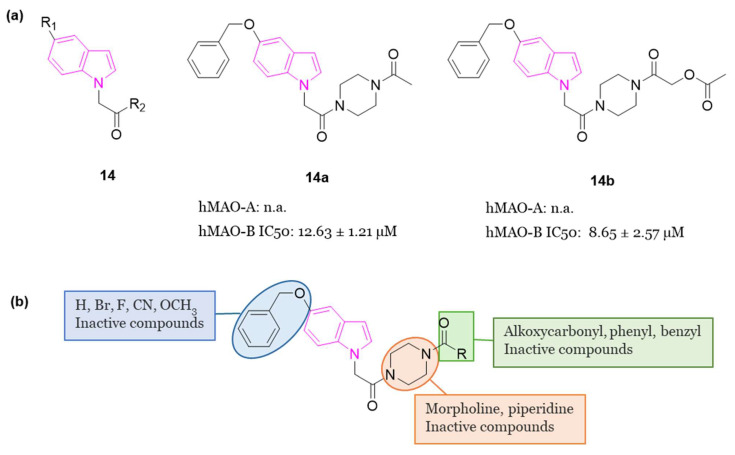
(**a**): General chemical structure of indole-based selective MAO-B inhibitors of series **14**, chemical structures and biological activities of compounds **14a** and **14b**; (**b**): SARs of indole-based selective MAO-B inhibitors of series **14**.

In 2022, Knez et al. developed 23 indole analogs (series **14**, Figure 9a), with the aim to identify reversible MAO inhibitors. Within the series, two compounds, **14a** (IC_50_ = 12.63 µM) and **14b** (IC_50_ = 8.65 µM), (Figure 9a), emerged as selective inhibitors of human MAO-B at low micromolar concentrations. Notably, these indoles exhibited comparable potency as the selective MAO-B inhibitor isatin (IC_50_ = 6.10 µM), but were less potent when compared to safinamide (Figure 2) (IC_50_ = 0.029 µM). SARs of this class of inhibitors are summarized in Figure 9b. Additionally, compounds **14a** and **14b** demonstrated favorable BBB permeation and low cytotoxicity in the human neuroblastoma cell line SHSY5Y, as assessed by MTS assay [53]. 

### 2.4. Adenosine A_2A_ Adenosine Receptor Antagonists

The A_2A_ adenosine receptor (A_2A_AR) is prominently present and shares proximity with the dopamine D_2_ receptor (D_2_R) in the striatum. Inhibiting the A_2A_AR could potentially counteract dopaminergic neurotransmission, particularly in relation to motor control in individuals with PD [54]. This potential impact of A_2A_AR antagonists has been substantiated through investigations involving rodent and primate PD models, along with initial clinical observations [55,56,57,58,59].

**Figure 10:**
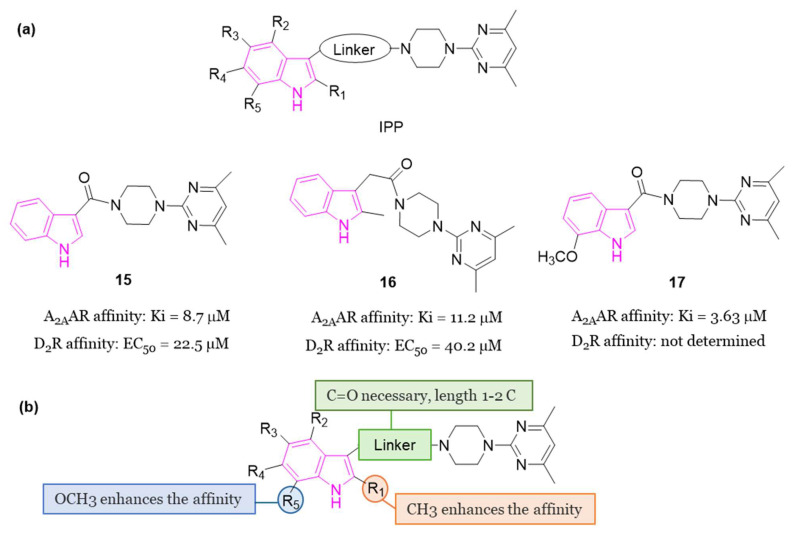
(**a**): General chemical structure of IPP-based A_2A_AR antagonists, chemical structures and biological activities of compounds **15**–**17**; (**b**): SARs of IPP-based A_2A_AR antagonists.

In 2018 [60], some compounds featuring the indole—piperazine–pyrimidine (IPP) scaffold (Figure 10a), with dual targeting capabilities for the human A_2A_AR and D_2_R, were developed. Among all IPPs developed, compounds **15** and **16** (Figure 10a) demonstrated affinity for the human (h) A_2A_AR in the radioligand competition assay (K_i_ = 8.7 and 11.2 μM for **15** and **16**, respectively) and for the hD_2_R in the artificial cell membrane assay (EC_50_ = 22.5 and 40.2 μM for **15** and **16**, respectively). Based on the obtained results, it was possible to derive some SARs delineating the important structural features of IPP-containing compounds for the hA_2A_AR binding (Figure 10b). The substitution at C2 of the indole scaffold with a methyl group (R_1_ = CH_3_) enhances the affinity for binding to hA_2A_AR. The linker connecting the C3 of the indole and the nitrogen of piperazine must incorporate a carbonyl moiety, and its length should be limited to one or two carbon atoms. Additionally, a methoxy group at C7 position of the indole nucleus (R_5_ = OCH_3_, compound **17**, Figure 10a) enhances the binding affinity to hA_2A_AR. Moreover, a functional test was performed to assess D_2_R activation with compound **16**. The results showed inhibition of cAMP accumulation in Chinese hamster ovary (CHO) cells at a concentration of 100 μM, comparable to the activity triggered by 100 nM of quinpirole, underscoring the D_2_R agonistic properties of compound **16**. Additionally, when compound **15** was subjected to further in vivo testing in the Drosophila model of PD at a concentration of 50 μM, it exhibited to produce enhanced movement and mitigation of the loss of dopaminergic neurons. Furthermore, in vitro toxicity studies for compounds **15** and **16** did not reveal any mutagenic effects up to 100 μM, nor hepatotoxicity or cardiotoxicity up to 30 μM [60]. 

### 2.5. PERK Signaling

**Figure 11:**
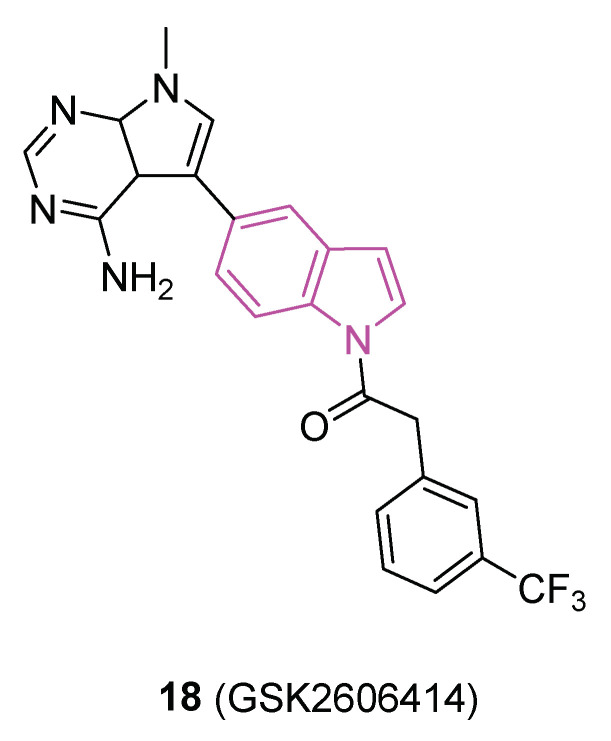
Chemical structure of PERK inhibitor **18** (GSK2606414).

Accumulating evidence indicates that disturbances in proteostasis are a notable characteristic of PD, with notable disruptions of the endoplasmic reticulum (ER), the primary compartment responsible for protein folding and secretion. Protein kinase R-like endoplasmic reticulum kinase (PERK) serves as a central sensor for ER stress, activating adaptive programs to restore homeostasis by halting protein translation and inducing the transcription factor ATF4. Prolonged PERK signaling, however, leads to apoptosis and neuronal dysfunction, attributed to the diminished translation of synaptic proteins. In 2018 [61], a study validated the activation of PERK signaling in postmortem brain tissue from PD patients and three distinct rodent models of the disease. Pharmacological intervention targeting PERK through oral administration of the indole compound **18** (GSK2606414, Figure 11) effectively suppressed the pathway in the substantia nigra pars compacta (SNpc) following experimental ER stress induction. **18** safeguarded nigral-dopaminergic neurons against a neurotoxin that induces PD, resulting in improved motor performance. The neuroprotective effects of PERK inhibition coincided with elevated dopamine levels and increased expression of synaptic proteins. Nevertheless, animals treated with **18** exhibited secondary effects potentially linked to pancreatic toxicity. This investigation suggests that strategies aimed at mitigating ER stress levels could prove effective in reducing neurodegeneration in PD.

### 2.6. AMPK Activators

The AMP-activated protein kinase (AMPK) serves as a crucial regulator of energy metabolism. This enzymatic complex, comprising AMPKα (catalytic), AMPKβ and AMPKγ (regulatory subunits), becomes activated in response to a reduction in ATP levels. AMPK activation hinders anabolic pathways while promoting catabolic pathways, working to restore energy equilibrium. Within brain physiology, AMPK functions as a metabolic sensor in the CNS, ensuring the preservation of energy homeostasis. Notably, it has been observed that pharmaceutical agents capable of activating AMPK exhibit a neuroprotective impact across various NDs [62]. 

An illustrative instance is metformin, showcasing its therapeutic efficacy in addressing conditions such as AD, PD, and HD [63]. 

Actually, HD patients with coexisting type 2 diabetes treated with metformin experienced an enhancement of cognitive functions, suggesting that the pharmacological stimulation of AMPK could serve as a viable approach in addressing HD [63,64].

**Figure 12:**
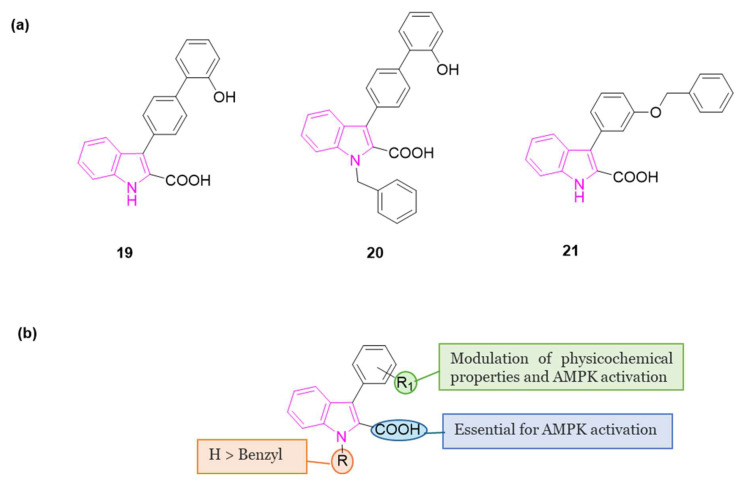
(**a**): Chemical structures of indole-based AMPK activators **19**–**21**; (**b**): SARs of indole-based AMPK activators.

In 2022 a series of indole-derived compounds was synthesized and evaluated for their capability to activate AMPK at the cellular level using HEK293 cell lines [65]. Within this series, compounds **19**–**21** (Figure 12a) exhibited a dose-dependent elevation in the phosphorylation of AMPKα when compared to untreated control cells, indicating the activation of AMPK. Notably, compound **21** emerged as the most potent activator of AMPK in this set of experiments.

During these preliminary investigations, exploration of the SARs unveiled that the presence of a carboxylic group at C2 of the indole ring is crucial for inducing AMPK activation in HEK293 cells. This was underscored by the lack of activity in compounds featuring an ester group at the same position (structures not reported). Conversely, full activity was observed in a similar structure with a carboxylic group at 2 position (**20**), reinforcing the significance of this structural element. For compounds **19** and **21**, both featuring a carboxylic group at C2, variations in the substitution pattern of aromatic residues at the C3 position influenced their physicochemical properties and accounted for observed differences in AMPK activation. Finally, indole nitrogen is generally unsubstituted, but substitution with benzyl group (**20**) is well tolerated to obtain AMPK activation (Figure 12b).

Simultaneously, alongside the AMPK activation assays, studies on cell viability were conducted on HEK293 cells. Compounds **19**–**21** exhibited minimal cell toxicity up to concentrations of 100 μM, with **21** being the least toxic compound, demonstrating no adverse effects even at 200 μM.

Furthermore, **21** exhibited a favorable in silico druggability profile, prompting its selection for subsequent investigations. Indeed, through the utilization of animal models for HD (both worms and mice), the in vivo effectiveness of **21** was validated, affirming its capacity to alleviate the neuropathological symptoms associated with this condition [65]. 

### 2.7. 5-HT_6_ Receptor (5-HT_6_R) Antagonists

**Figure 13:**
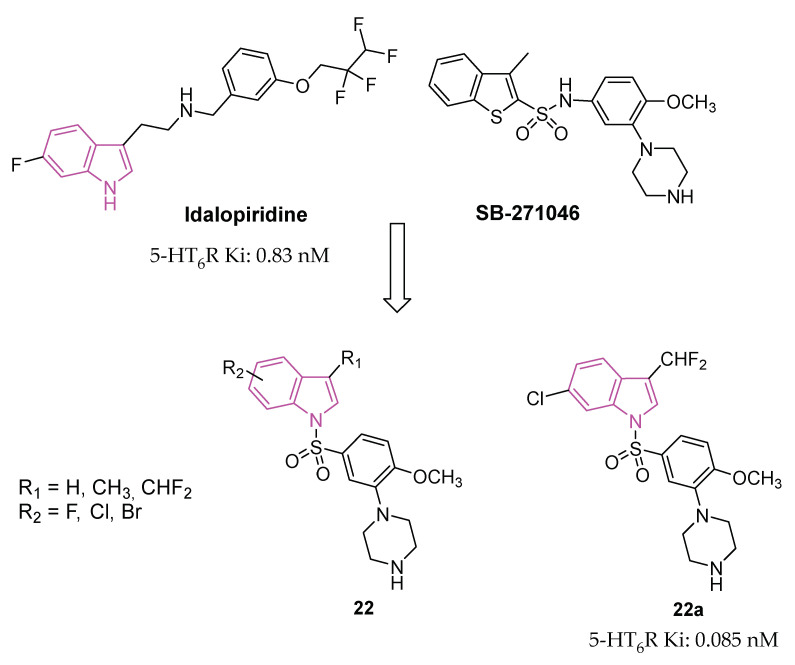
Design and general chemical structure of indole-based 5-HT_6_R antagonists of series **22**, chemical structure and biological activity of compounds **22a**.

The serotonin type 6 receptor (5-HT_6_R) is a G protein-coupled receptor (GPCR) resulting as a promising target for addressing cognitive deficits in neurodegenerative and psychiatric disorders. Its expression is predominantly limited to CNS, with a focus on key brain regions associated with cognitive functions such as the prefrontal cortex, hippocampus, and striatum. Preclinical models, ranging from new object and social recognition to spatial memory tasks, have consistently shown that 5-HT_6_ receptor antagonists improve learning and memory [66]. The mechanism by which blocking the 5-HT_6_ receptor has pro-cognitive effects is still uncertain. One hypothesis is that there is interaction with the cholinergic system since the treatment with idalopirdine (Figure 13), a 5-HT_6_ receptor antagonist, gave a synergistic effect in improving cognition with an AChE inhibitor in both animal models and AD patients [67]. In addition, neurophysiological and neurochemical tests have confirmed the synergistic effect of this drug combination on cholinergic function [68]. However, recent data show that cholinergic neurons do not express 5-HT_6_ receptors, highlighting that this is an indirect mechanism [67,69]. 

Anyhow, antagonists of 5-HT_6_R exhibit positive cognitive effects, as demonstrated not only by various preclinical but also by initial clinical studies, indicating pro-cognitive benefits in humans [70,71,72,73].

Several 5-HT_6_R antagonists, including idalopirdine and intepirdine (Figure 13), have shown statistically significant improvements in cognitive functions, when used as add-on therapies to donepezil compared to donepezil alone. Additionally, preclinical studies have linked 5-HT_6_R to cognitive dysfunction, affective disorders, anxiety, and depression. Consequently, 5-HT_6_R antagonists are considered potential candidates for AD therapy [74,75,76,77,78]. 

In the pursuit of more efficient 5-HT_6_R antagonists, the structural characteristics of two well-known antagonists, idalopirdine, and SB-271046 (Figure 13), were investigated in 2022 [79]. The 6-fluoro-1H-indole moiety of idalopirdine and the 1-(2-methoxyphenyl)piperazine fragment of SB-271046 were particularly considered to afford compounds of series **22** (Figure 13). 

Further SAR studies focused on modifying the C3 and C6 positions of the indole core to enhance 5-HT_6_R binding potencies. Various halogen groups (F, Cl, Br) and different groups (Me, CHF_2_) were introduced at the C5 and C6 positions and the C3 position of the indole ring, respectively. These modifications led to the identification of the most promising compound, **22a** (Ki = 0.085 nM), exhibiting a 10-fold higher affinity at 5-HT_6_R than idalopirdine (Ki = 0.83 nM). Apart from its cognition-enhancing properties, **22a** efficiently reversed scopolamine-induced emotional memory deficits, demonstrating favorable pharmacokinetic and in vitro metabolic properties.

## 3. Multifunctional Indole-Based Compounds as Potential Anti-Neurodegenerative Agents

NDs are complex conditions involving a network of interconnected factors and a multifactorial pathomechanism. Therefore, effective treatment strategies for NDs may benefit from the use of multifunctional compounds [80,81,82,83,84,85].

**Figure 14:**
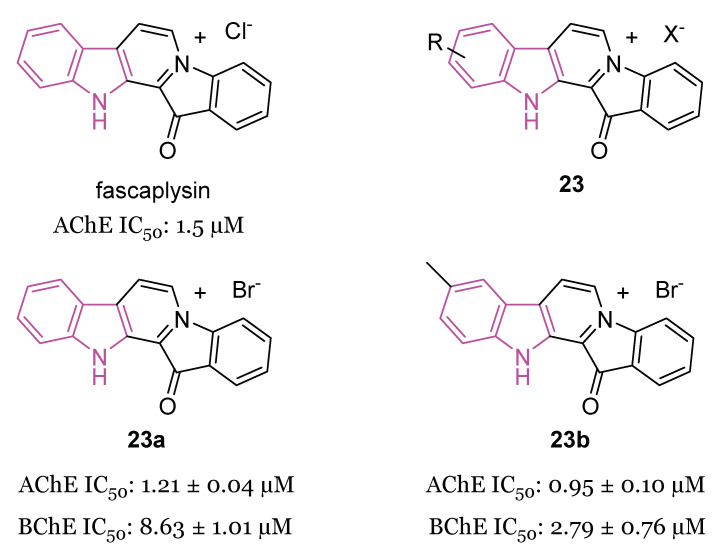
Chemical structure of fascaplysin, general chemical structure of indole-based derivatives of series **23**, chemical structures and biological activities of compounds **23a** and **23b**.

Fascaplysin, (Figure 14) a β-carboline alkaloid, was reported to inhibit AChE with IC_50_ ≅ 1.5 μM [86], and, recently, to prevent Aβ aggregation and protect against Aβ oligomer-induced neuronal death [87], so possessing anti-AD properties.

In 2019, a series of fascaplysin derivatives **23** (Figure 14) were synthesized and evaluated in vitro for their ChE inhibition activities, their neuronal protective effects, and toxicities [88]. The most potent compounds in vitro, **23a** and **23b** (Figure 14), which could effectively reduce neurotoxicity (H_2_O_2_-induced) in the nanomolar range, were selected to evaluate their cognitive-enhancing effects in animals. Both compounds **23a** and **23b** demonstrated improvement of cognitive impairment caused by scopolamine or Aβ oligomers in mice, without impacting locomotor functions, preventing cholinergic dysfunctions, reducing the expression of pro-inflammatory cytokines, and inhibiting Aβ-induced tau hyperphosphorylation in vivo. Crucially, pharmacodynamics studies indicated that **23b** has the capability to penetrate the BBB and to be retained in the CNS, resulting in lower in vivo acute toxicity compared to fascaplysin [88]. 

**Figure 15:**
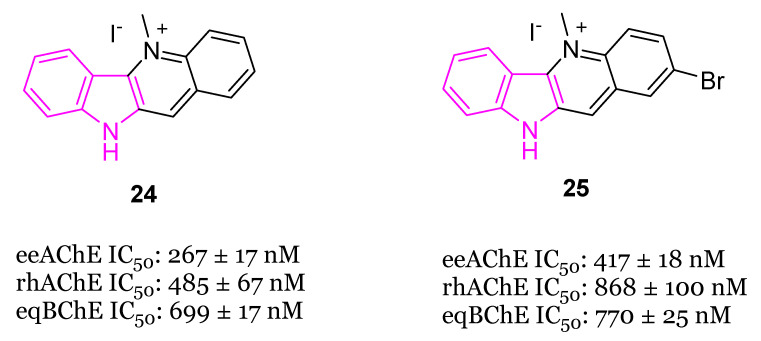
Chemical structures of cryptolepine (**24**) and 2-bromocryptolepine (**25**).

In the same year [89], the synthesis and biological evaluation of indoloquinoline alkaloid cryptolepine (**24**) and its 2-bromo-derivative (**25**, Figure 15) as dual inhibitors of AChE and BChE were reported. In particular, cryptolepine (**24**) inhibited eeAChE, rhAChE and eqBChE with IC_50_ values of 267, 485 and 699 nM, respectively, resulting more potent of its 2-bromo-derivative **25** (IC_50_ values of 415, 868 and 770 nM, respectively). The mechanism of action of compounds **24** and **25**, revealed through kinetic studies, consists of a non-competitive ChEs inhibition. Moreover, compounds **24** and **25** were able to stop the formation of toxic Aβ oligomers, through the BACE-1 inhibition, and to increase the Aβ clearance, through the P-glycoprotein (P-gp) induction. In particular, cryptolepine (**24**) displayed potent P-gp induction activity at 100 nM, in P-gp overexpressing adenocarcinoma LS-180 cells and an excellent toxicity window in this cell line and also in human neuroblastoma SH-SY5Y cell line. Molecular modeling studies evinced that compounds **24** and **25** were tightly packed inside the active site gorge of both ChEs via multiple π-π and cation-π interactions. Moreover, both compounds established hydrophobic interactions with the allosteric PAS. Finally, the ADME properties of the two compounds, including the BBB permeability, resulted within the acceptable range of values [89]. 

**Figure 16:**
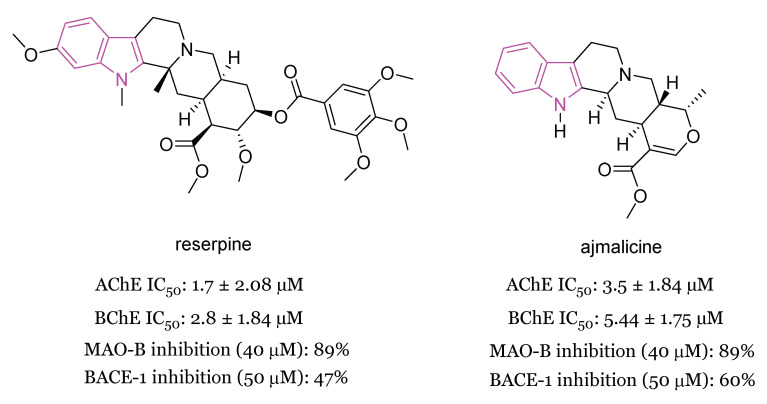
Chemical structures and biological activities of reserpine and ajmalicine.

In 2020 [90] the MTDL potential of the indole alkaloids reserpine and ajmalicine (Figure 16) (major secondary metabolites of Rauwolfia serpentina) in AD has been highlighted. In Aβ_42_ samples reserpine and ajmalicine reduced the percentage of β sheet content showing their anti-amyloidogenic potential which also resulted to be concentration-dependent. The mechanism of inhibition was understood through molecular docking analysis, which evidenced that reserpine and ajmalicine stack between adjacent β sheets, thus inhibiting further oligomerization. Moreover, reserpine and ajmalicine counteracted the decrease in AChE levels in vitro by significantly inhibiting BChE in a concentration dependent manner. Docking studies also suggested a stable protein–ligand complex between reserpine and ajmalicine with BChE.

In vitro assays, complemented with molecular docking studies, demonstrated that reserpine and ajmalicine also significantly inhibited the beta-site APP cleaving enzyme 1 (BACE-1) with ajmalicine being a more potent inhibitor than reserpine. Finally, reserpine and ajmalicine have been shown to inhibit also MAO-B enzyme in a concentration dependent manner, with ajmalicine giving a higher binding score than reserpine. The MTDL potential and the ADMET profile of ajmalicine make this compound able to act as ideal drug molecule with BBB permeability in relieving symptoms and being potentially effective in AD [90].

**Figure 17:**
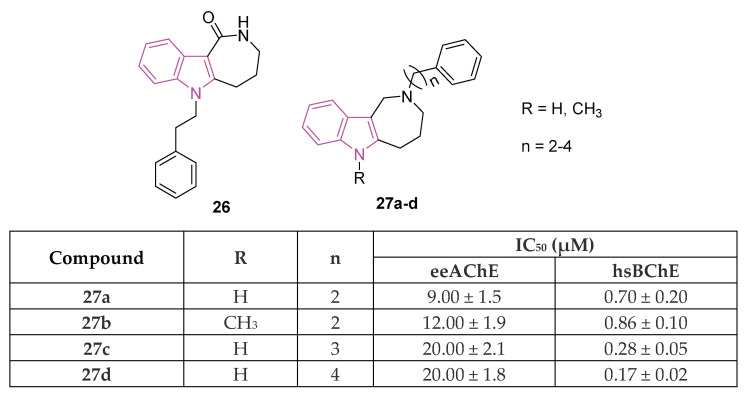
Chemical structures and biological activities of indole-based derivatives **26** and **27a**–**d**.

Azepino indoles have been demonstrated to serve as binding components in various dual-site AChE inhibitors and MTDLs targeting the pathological pathways of NDs. Specifically, the 2,3,4,5-tetrahydroazepino[4,3-b]indole(1H)-2-one derivative **26** (Figure 17), showed potent nanomolar inhibition of BChE with selectivity towards AChE (IC_50_ against AChE and BChE being 20 and 0.020 μM, respectively). Additionally, it displayed protective effects against NMDA-induced excitotoxicity in a neuronal cell line (SH-SY5Y) [91]. Regrettably, **26** has limited solubility in water and its conversion to the more soluble amino derivative leads to a reduction in BChE inhibition effectiveness and to an increase in toxicity towards neuronal cells. Recently, in order to gain a better understanding of the azepino[4,3-b]indole nucleus as a potential framework for new MTDLs aimed at treating AD, approximately twenty N2-substituted 1,2,3,4,5,6-hexahydroazepino[4,3-b]indole derivatives were synthesized and assessed in vitro as inhibitors of ChEs and amyloid Aβ aggregation [92]. SAR investigation, primarily focused on substituting the azepine nitrogen with groups of varying size and lipophilicity, led to the discovery of N2-phenylalkyl derivatives **27a**–**d** (Figure 17) as inhibitors of human serum BChE (hsBChE) at submicromolar concentrations, with selectivity ranging from ten to a hundred-fold over eeAChE. 

The most potent compound, the N2-phenylbutyl derivative **27d**, acted as a mixed-type inhibitor, exhibiting in vitro inhibitory activity (with an IC_50_ of 0.2 μM) and selectivity (>100-fold) against hBChE. Molecular docking simulations of compound **27d** within the hBChE binding site suggested that π-stacking interactions involving Trp82 and Trp231, along with two hydrogen bonds with the Thr120 side chain and His438 carbonyl backbone, were crucial for its inhibitory activity. Moreover, compound **27d** displayed significant protective effects against cytotoxicity induced by Aβ_42_ peptide and oxidative stress in the neuroblastoma SH-SY5Y cell line. This makes it a promising candidate for further molecular optimization and in vivo pharmacological studies, aimed at evaluating its potential as MTDL for the management of NDs [92]. 

The dual inhibition of fatty acid amide hydrolase (FAAH) and cholinesterases (ChEs) has recently egressed as a novel strategy for the management of neurodegeneration [93]. 

The exploration of the involvement of the endocannabinoid system (ECS) and fatty acid amide hydrolase (FAAH) in neurodegenerative processes has become an intriguing area of study. Boosting ECS functionality by locally or globally inhibiting the FAAH enzyme holds promise for addressing a range of inflammatory and neuropathic pain-related disorders. In the initial phases of AD, preventing the deactivation of ECS was discovered to diminish Aβ-induced gliosis, neuronal death, and memory loss. Investigations have unveiled changes in endocannabinoid signaling in diverse NDs, including AD, where a reduction in anandamide levels resulted in the generation of Aβ. In the context of AD-related inflammatory processes, microglia and astrocytes exhibit heightened FAAH expressions. Given that anandamide is a substrate for FAAH, increased enzyme concentration in astrocytes around neuritic plaques reduces anandamide, diminishing neuroprotection. Consequently, inhibiting FAAH becomes a potential strategy to prevent or alleviate inflammatory processes linked to Aβ deposition by indirectly elevating endocannabinoid signaling to therapeutic levels [94,95].

**Figure 18:**
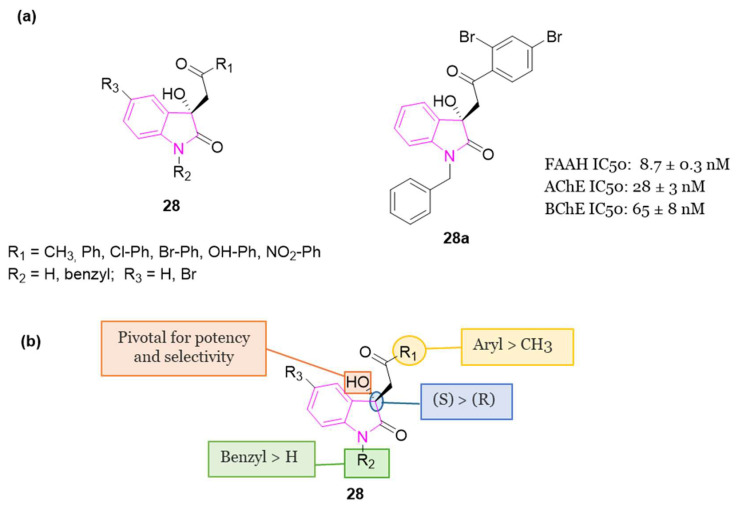
(**a**): General chemical structure of indole-based derivatives of series **28**, chemical structure and biological activity of compound **28a**; (**b**): SARs of indole-based derivatives of series **28**.

In 2020 [96], a library of 3-hydroxy-3-phenacyloxindole derivatives **28** (Figure 18) incorporating a short acetyl linker was evaluated for FAAH and ChEs (AChE/BChE) inhibition. 1-Benzyl-3-hydroxy-3-(2′,4′-dibromophenacyl)oxindole **28a** (Figure 18) resulted the most promising compound, showing a balanced multifunctional profile with FAAH (IC_50_ = 8.7 nM), AChE (IC_50_ = 28 nM), and BChE (IC_50_ = 65 nM) inhibition. SAR studies predicted multifarious aspects crucial for potency of these analogs indicating that, in general, N-benzyl isatin analogs are more potent than 5-bromoisatin- and isatin-based ones. The structural geometry and rigidity conferred by the oxindole portion reinforce the adhesion of the compounds within the binding pockets of the target enzymes. Studies on the alignment of test inhibitors within FAAH, AChE, and BChE revealed that two aryl moieties are crucial for achieving optimal binding and stabilization of the inhibitors within the active sites of the respective enzymes. It is essential for one of these aryl binding sites to consist of a sizable, rigid component with both H-bond acceptor and donor groups. Furthermore, the presence of a less flexible, short linker guiding both aryl binding moieties into the enzymatic cavity, along with the 3-OH group, is pivotal for enhancing their potency and selectivity. Examination of the binding interactions and conformations of stereoisomers of the primary inhibitors underscored the significance of (S)-stereochemistry at the C-3 position of the oxindole scaffold in terms of both potency and selectivity. Compound **28a** also exhibited antioxidant capabilities and demonstrated to be non-neurotoxic. Finally, in silico assessments of molecular and ADMET properties predicted drug-like characteristics for the test compounds, suggesting their suitability for oral administration [96].

**Figure 19:**
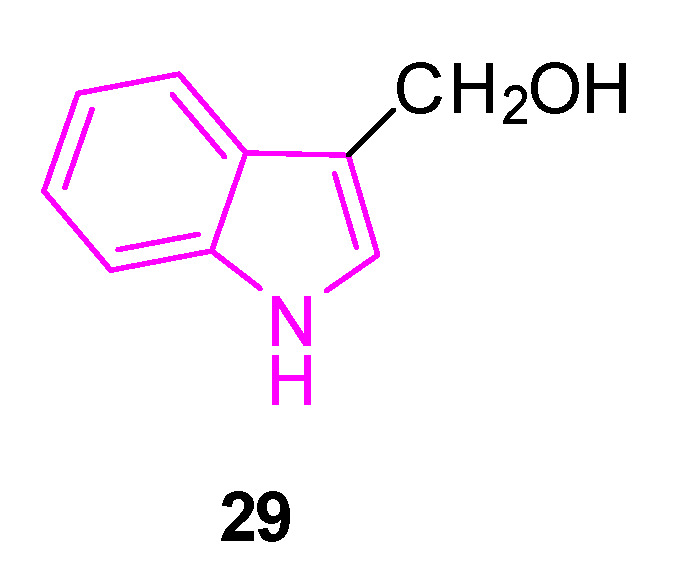
Chemical structure of indole-3-carbinol **29**.

The same year, a research study was conducted to investigate the impact of inhibitors targeting nuclear factor κB (NF-κB) on neurobehavioral abnormalities and neuroinflammation in PD [97]. The study employed cost-effective in silico methods, utilizing docking-based ligand-target complex predictions and assessing optimal physicochemical properties to identify a lead NF-κB inhibitor from a database. The investigations identified indole-3-carbinol (**29**, Figure 19) as a potential hit, which was then considered for subsequent pharmacological validations.

**29** is a compound produced by β-thioglucosidase-mediated autolysis of glucobrassicin from consumption of cruciferous vegetables, which has been extensively researched for its potential protective properties. Notably, it has demonstrated significant neuroprotective activity in a rat model of depressive behavior induced by clonidine. Likewise, compound **29** has shown a pronounced protective effect against injury caused by glutamate excitotoxicity in an in vitro model of cerebral ischemia [97]. 

In the study of 2020, to simulate neuroinflammation in PD, lipopolysaccharide (LPS) was intranigral administered to rats as a model [97]. This induced impairment in motor functions, coordination, learning, and memory, as evidenced by tests such as the rotarod apparatus, beam balance test, open field test, and Morris water maze test. Chronic administration of compound **29** for 21 days in LPS-treated rats significantly improved motor functions, coordination, learning, and memory. These improvements were associated with a decrease in the activity of inflammatory cytokines such as TNF-α and IL-6. Additionally, **29** was found to inhibit NF-κB, whose levels had increased after LPS administration. Furthermore, administration of compound **29** in LPS-treated rats resulted in decreased levels of malondialdehyde and increased levels of reduced glutathione, superoxide dismutase, and catalase in the cortex and striatum. These findings suggest a potential neuroprotective effect of **29** by mitigating LPS-induced behavioral alterations, oxidative damage, and neuroinflammation. This effect is attributed to the potent antioxidant and anti-inflammatory (NF-κB inhibition) properties of **29**. Notably, the impact of compound **29** at a dose of 50 mg/kg was comparable to that deriving from levodopa-carbidopa combination (LD:CD). Moreover, combining **29** (50 mg/kg) with LD:CD exhibited a synergistic effect in improving motor impairments and cognitive deficits [97]. 

Interestingly, compound **29** has the potential to activate sirtuin 1 (SIRT1), a highly conserved protein that plays a crucial role not only in the normal aging process of the brain, but also in improving various NDs in animal models [98].

In 2022, a study was conducted to explore the neuroprotective effects of compound **29** against rotenone (ROT)-induced PD in male albino rats [99]. PD was induced through subcutaneous administration of ROT (2 mg/kg) over a period of 28 days. The activity of **29** at doses of 25, 50, and 100 mg/kg/day was evaluated using the catalepsy test (bar test), spontaneous locomotor activity, rotarod test, weight change, expression of tyrosine hydroxylase (TH) and α-synuclein (α-Syn), striatal dopamine content, and histological examination.

The highest dose of **29** (100 mg/kg) demonstrated the most significant effectiveness in preventing ROT-induced motor dysfunctions. It also mitigated striatal dopamine decrease, weight loss, neurodegeneration, reduction in TH expression, and increase in α-Syn expression in both the midbrain and striatum. Further mechanistic investigations revealed that the neuroprotective effects of **29** can be partially attributed to its anti-inflammatory and antiapoptotic properties, as well as the activation of the SIRT1/AMPK pathway [99].

**Figure 20:**
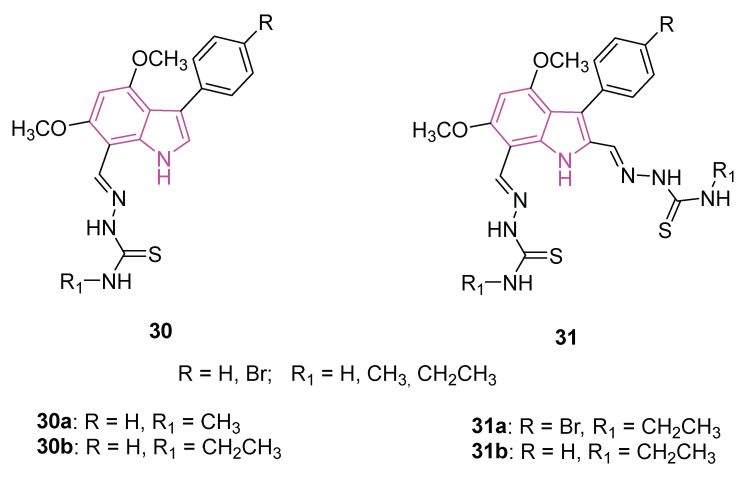
Chemical structures of monomeric (**30a**, **30b**) and dimeric (**31a**, **31b**) 3-substituted 4,6-dimethoxyindoles-based thiosemicarbazones.

In the same year, two series of monomeric (**30**) and dimeric (**31**) 3-substituted 4,6-dimethoxyindol-based thiosemicarbazones (Figure 20) were synthesized and assessed for their potential to target ChEs [100]. Thiosemicarbazones represent a significant class of compounds renowned for their diverse biological activities [101], as the conjugated N-N-S system imparts a significant affinity for metal ions such as iron, zinc, and copper leading to the formation of metal complexes, serving as crucial biological targets involved in interactions with nucleic acids [102,103,104]. Moreover, the thiosemicarbazone moieties present a valuable resource for forming hydrogen bonds with the relevant amino acids located in the ester or acyl binding pockets of ChEs. The presence of an additional thiosemicarbazone (series **31**) carrying distinct residues may play a crucial role in fostering additional hydrophobic and hydrogen bond interactions, thereby enhancing potential interactions. 

The biological significance of the two series, **30** and **31**, was explored through evaluations involving AChE and BChE enzymes (galantamine as positive control), coupled with three distinct antioxidant property determination assays, namely DPPH free radical scavenging, ABTS cationic radical decolorization, and CUPRAC cupric reducing antioxidant capacity (BHA, BHT, α-tocopherol as reference compounds). Compounds **30a** and **30b** exhibited notable inhibitory activity against BChE, with IC_50_ values of 7.42 and 1.95 μM, respectively (Table 1). While the antioxidant potentials were deemed moderate for DPPH and ABTS assays, compounds **31a** and **30b** emerged as the most potent candidates in both antioxidant assessments (Table 1). In the cupric reducing capacity assay, compounds **31a** and **31b** displayed superior inhibition values compared to all standards. Further insights into binding modes and affinities were gained through molecular docking and molecular dynamics simulations, aligning with the experimental observations [100]. 

Finally, in in silico studies, compounds **30a** and **30b** showed BBB permeability scores indicating a good penetration into the brain, while all other compounds of series **30** and **31** had a score indicating poor BBB permeability.

As a result, compound **30b** is the most plausible candidate that can compete with galantamine and deserves further study. 

**Figure 21:**
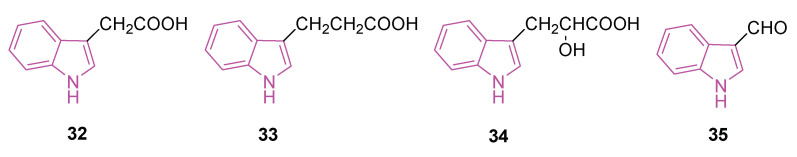
Chemical structures of indole-3-acetic acid (**32**), indole-3-propionic acid (**33**), indole-3-lactic acid (**34**), and indole-3-carboxyaldehyde (**35**).

Recently, mounting evidence suggests that indole and its derivatives are significantly involved in the cognitive decline associated with aging [105]. In a study conducted in 2021, it was disclosed that dietary Trp intervention may enhance the GPCR30GPR30/5’-adenosine monophosphate (AMP)-AMPK/SIRT1 pathway in the colon and brain of aging mice. This intervention resulted in elevated levels of indoles, particularly indole-3-acetic acid (**32**) and indole-3-propionic acid (**33**) (Figure 21), aiming to counteract the decline of the levels of indoles in colon content observed in aging mice [106]. Therefore, a hypothesis emerged suggesting that Trp or its metabolites could potentially counteract neurodegeneration through a unique mechanism involving the activation of GPR30, consequently influencing the AMPK pathway during the aging process.

In 2023 [107], a study was conducted to assess the protective mechanisms of Trp metabolites against neurodegeneration during the aging process. To achieve this, the neuroprotective qualities of Trp metabolites were investigated using both in vitro and in vivo experimental models. Trp metabolites, including indole, indole-3-acetic acid (**32**), indole-3-propionic acid (**33**), indole-3-lactic acid (**34**), and indole-3-carboxyaldehyde (**35**) (Figure 21), exhibited significant reductions in oxidative stress, inflammation, and neuronal apoptosis induced by H_2_O_2_ in HT-22 cells. Simultaneously, indoles upregulated the expressions of the (GPR30)/AMPK/SIRT1 pathway in vitro. 

Moreover, the neuroprotective effects of **32** and **33** were demonstrated through the activation of the GPR30/AMPK/SIRT1 pathway in vivo, particularly in d-galactose-induced aging mice. Lastly, the regulatory impact of indoles produced by gut microbiota on the GPR30/AMPK/SIRT1 pathway was further substantiated by pretreating HT-22 and Neuro-2a cells with a GPR30 antagonist. In this scenario, indoles were validated for their inhibitory effects on neurodegeneration by activating the GPR30/AMPK/SIRT1 pathway during the aging process.

**Figure 22:**
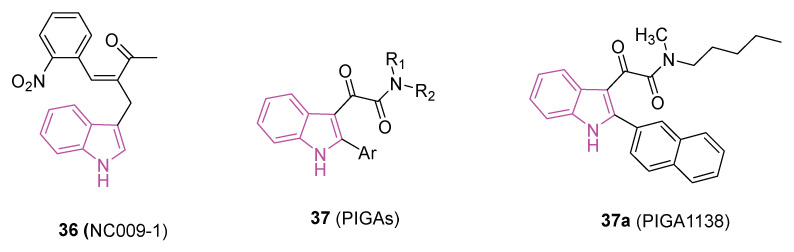
Chemical structures of compounds **36** (NC009) and **37a** (PIGA1138), general chemical structures of phenylindolylglyoxylamides (PIGAs) **37**.

Recently, indole compound **36** (NC009-1, Figure 22) has demonstrated the capability to reduce Aβ-aggregation and provide neuroprotective effects through the activation of heat shock protein beta 1 (HSPB1) in a cell model of tauopathy [108], in spinal spinocerebellar ataxia (SCA) type 17 cells, and in SCA17 mouse models [109]. 

Moreover, compound **36** triggered the activation of apolipoprotein E (APOE) and neurotrophic receptor tyrosine kinase 1 (NTRK1) in Aβ-GFP SH-SY5Y cells. It also exhibited this activation in vivo in Aβ precursor protein (APP)/presenilin 1 (PS1)/microtubule-associated protein tau (Tau) triple transgenic (3×Tg-AD) mouse models of AD [110]. Simultaneously, it diminished the IL-1β-mediated pathway in the SCA type 3 SH-SY5Y cell model inflamed with IFN-γ-primed HMC3 conditioned medium [110]. In a 2023 study, researchers explored the neuroprotective capabilities of NC009-1 in MPP^+^-activated human microglial HMC3 cells and/or the sub-chronic MPTP-induced mouse model of PD [111]. The findings revealed that **36** displays neuroprotective properties through the modulation of inflammatory and anti-oxidative pathways. Importantly, it should be highlighted that **36** exhibited favorable bioavailability and the potential to penetrate BBB [109], thereby bolstering its prospects for clinical translation.

Multiple sclerosis (MS) is a condition affecting the CNS, characterized by autoimmune responses leading to demyelination, neuroinflammation, and neuronal dysfunction due to peripheral immune cell infiltration. While treatments exist for relapsing-remitting MS (RRMS), there remains a lack of therapies for primary progressive MS (PPMS).

Recent research indicates a potential therapeutic role for the steroidogenic 18 kDa translocator protein (TSPO) in managing neuroinflammation and demyelination; this has led to evaluation of TSPO ligands as potential treatments for NDs [112]. In particular, phenylindolylglyoxylamides **37** (PIGAs, Figure 22), have shown neuroprotective effects by stimulating steroidogenesis gaining pro-survival properties against cytotoxic insults and inflammatory responses [113,114]. 

In a recent study [115], the therapeutic potential of **37**, particularly **37a** (PIGA1138, Figure 22), was investigated in an experimental autoimmune encephalomyelitis (EAE) mouse model of PPMS. PIGA1138 (15 mg/kg) significantly reduced disease severity, improved motor function, preserved myelin integrity, and prevented axonal damage in the CNS. Additionally, it inhibited immune cell infiltration and promoted anti-inflammatory responses, suggesting its potential in managing PPMS [115,116]. 

## 4. Neuroprotective Effects of Multifunctional Indole Hybrid Compounds

The incorporation of the indole moiety with diverse bioactive components has been employed in the development of MTDLs useful in neurodegeneration. The following section provides an updated overview (2018–2023) of indole hybrid compounds, some of which are described elsewhere [25,117], and their prospective contributions to the therapeutic landscape for NDs.

### 4.1. Indole Hybrids as Cholinesterase Inhibitors and/or Antioxidants

#### 4.1.1. Pyrido[3,4-b]indoles (β-Carbolines) Hybrids

**Figure 23:**
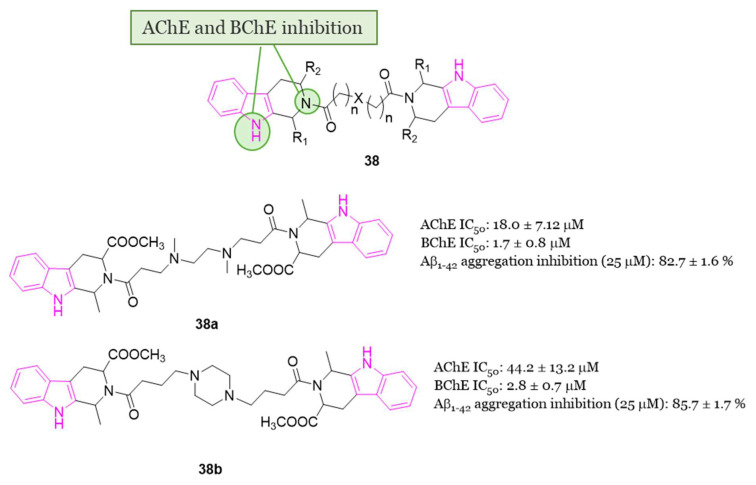
SARs of 1,2,3,4-tetrahydro-β-carboline hybrids of series **38**, chemical structures and biological activities of compounds **38a** and **38b**.

The β-carbolines (pyrido[3,4-b]indoles) are recognized for their inhibitory effects on AChE, BChE, and MAO-B [25]. Due to solubility issues associated with the hydrophobic component of β-carboline, in 2018 Zhao et al. conducted a study exploring the multifunctional properties of bivalent 1,2,3,4-tetrahydro-β-carboline derivatives **38** (Figure 23) [118]. In an effort to address solubility concerns, two 1,2,3,4-tetrahydro-β-carboline moieties were conjugated via a linker featuring a hydrophilic nature, such as a piperazine and ethylendiamine moiety. Additionally, the nitrogen atom within the linker has the potential to engage in a cation-π interaction with the amino acids located in the pocket of the ChE enzyme, so possibly enhancing the inhibitory activity of the compounds. All compounds of series **38** (Figure 23) exhibited promising multifunctional properties with N,N′-dimethyl-bis(1-(1-methyl-1,2,3,4-tetrahydro-β-carboline-3-carboxylic acid methyl ester)propan-1-one)-ethylenediamine **38a** (Figure 23) and bis(1-(1-methyl-1,2,3,4-tetrahydro-β-carboline-3-carboxylic acid methyl ester)butan-1-one)-piperazine **38b** (Figure 23) demonstrating exceptional potency and selectivity for eeBChE (IC_50_ = 1.7 and 2.8 μM, respectively) over eeAChE (IC_50_ = 82.7 and 85.7 μM, respectively). Both compounds **38a** and **38b** also exhibited significant inhibition of Aβ_1-42_ aggregation (82.7% and 85.7%, respectively, at 25 μM). Molecular docking studies on BChE revealed that **38a** was engaged in π-π stacking and π-alkyl interactions with Trp82, forming two hydrogen bonds with Thr284 and His438 residues. Similarly, **38b** demonstrated favorable interactions with the enzyme BChE [118]. Moreover, compounds **38a** and **38b** displayed antioxidant activity and a neuroprotective effect. In fact, both compounds exhibited almost 1.5-fold higher inhibition of Aβ_1-42_ aggregation than resveratrol, without inducing cytotoxicity in SH-SY5Y cells at concentrations ranging from 1 to 5 µM in an MTT assay. More interestingly, **38a** and **38b** provided protection against okadaic acid- and H_2_O_2_-induced neurotoxicity (oxidative stress/cell damage) in SH-SY5Y cells [118]. 

**Figure 24:**
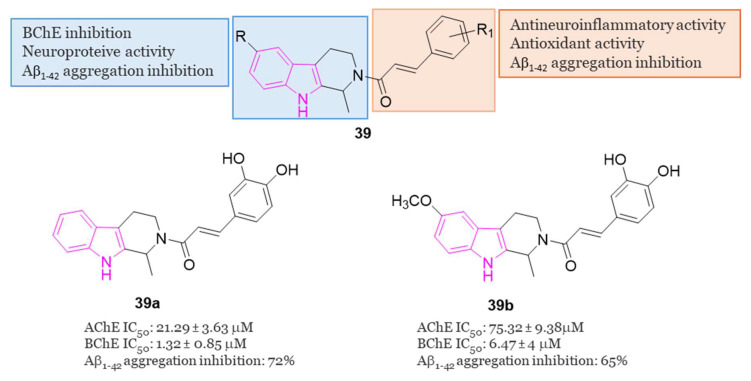
General chemical structure and SARs of 1,2,3,4-tetrahydro-β-carbolines hybrids of series **39**, chemical structures and biological activities of compounds **39a** and **39b**.

In 2020 [119], Liao et al. conducted a study exploring the potential of hybrids combining 1,2,3,4-tetrahydro-β-carboline with cinnamic acid, as cinnamic acid is a natural product well known for preventing neuro-inflammation, acting as an antioxidant, and inhibiting amyloid aggregation [25]. The researchers aimed to enhance the physicochemical properties of 1,2,3,4-tetrahydro-β-carboline while reducing its toxicity in hybrid molecules of general formula **39** (Figure 24) [119]. Among the compounds of this series, **39a** and **39b** (Figure 24) stood out as the most promising, displaying 72.5% and 65.5% inhibition of Aβ_1-42_ aggregation at 25 µM, respectively. Both compounds, featuring 3,4-dihydroxy groups at the phenyl ring, outperformed those with a methoxy group, suggesting that a conjugated system with polyhydroxyl groups is optimal for inhibiting Aβ_1-42_ aggregation. Molecular docking studies revealed that compound **39a** formed hydrophobic and hydrogen-bonding interactions in the β-sheet of the amyloid-forming peptide. Additionally, both **39a** and **39b** exhibited greater selectivity for BChE (IC_50_ = 6.47 and 1.32 µM) over AChE (IC_50_ = 75.3 and 21.3 µM). In-depth analysis indicated that the carboline core of **39a** interacted with Tyr332 of BChE through π-π stacking, the N atom of the indole ring formed a hydrogen bond with Asp70, and the phenyl ring of the cinnamic acid moiety interacted with Trp82 via π-π interactions. Furthermore, two OH groups on the aromatic ring of cinnamic acid formed two hydrogen bonds with Gly115 residue. Compounds **39a** and **39b** demonstrated non-neurotoxic effects, showing no cytotoxicity in PC12, SHSY5Y, BV-2, HT22, and L02 cell lines. Both compounds protected against H_2_O_2_-induced cell damage, okadaic acid-induced cytotoxicity, and Aβ_1–42_-induced cell toxicity in human PC12 and SH-SY5Y cells. Additionally, they reduced LPS-induced ROS production in BV2 cells. Finally, in an AD mice model, orally administered **39a** and **39b** restored learning and memory function to a level comparable to that of the control. Importantly, they did not exhibit any acute toxicity in vivo even at a relatively high dose of 600 mg/kg.

#### 4.1.2. Indole-Tacrine Hybrids

**Figure 25:**
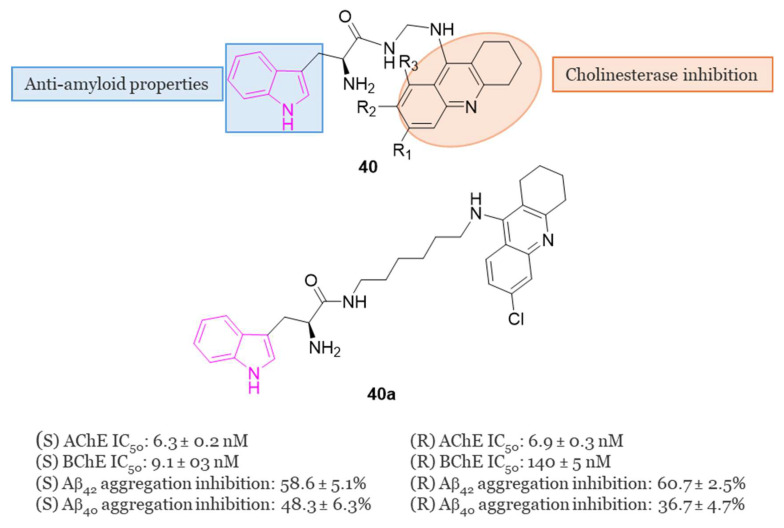
General chemical structure of indole-tacrine hybrids of series **40**, chemical structure and biological activity of compound **40a**.

In 2019, Chalupova et al. developed a series of hybrid derivatives combining tacrine and L-tryptophan to obtain compounds of series **40** (Figure 25) [120]. Neprilysin, produced in the brain, is a zinc-dependent metalloproteinase enzyme that aids in Aβ clearance. Tryptophan metabolites, specifically 5-hydroxyindole-acetic acid (5-HIAA) and kynurenic acid (KYNA), have been identified as regulators of both the activity and the expression of neprilysin in the brain under normal and pathological conditions such as AD [121]. Consequently, therapies based on tryptophan derivatives, or its metabolites may contribute to reducing amyloid protein accumulation in the brains of AD patients.

Among all the series, SK-1035 (**40a**) with a six-methylene long linker, exhibited the highest inhibitory activity against both ChEs (IC_50_ = 6.3 nM for hAChE and 9.1 nM for hBChE). It demonstrated inhibition of Aβ_42_ self-aggregation (58.6% at 50 µM) and hAChE-induced Aβ_40_ aggregation (48.3% at 100 µM). The S-enantiomer was found to be 15 times more potent as an hBChE inhibitor than the R-enantiomer (IC_50_ = 9.1 nM vs. 140 nM, respectively). X-ray studies revealed compound **40a** as the most potent hybrid heterodimer, interacting with both the CAS and PAS of AChE. Subsequent in vitro assessments indicated that this compound could penetrate the BBB and exhibited moderate neuronal nitric oxide inhibitory activity, along with improvement in scopolamine-induced cognitive deficit in experimental animals [120].

#### 4.1.3. Indole–Spiropyrrolidine Hybrids

**Figure 26:**
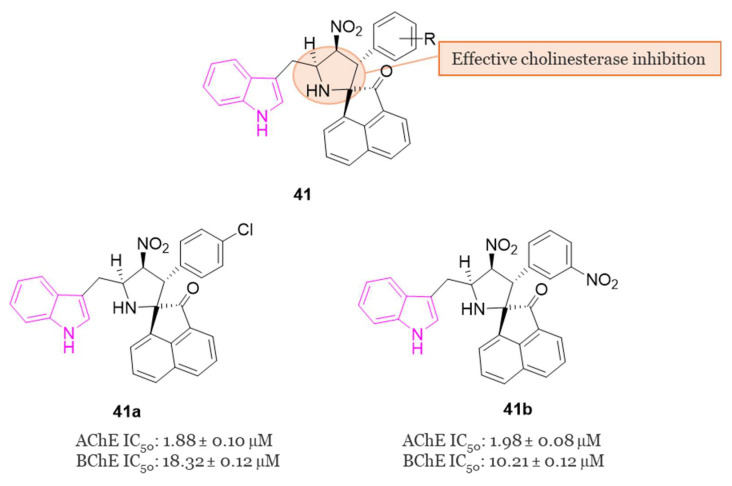
General chemical structure of indole–spiropyrrolidine hybrids of series **41**, chemical structures and biological activities of compound **41a** and **41b**.

In 2018, Arumugam et al. conducted a study examining the ChE inhibitory activities of spiropyrrolidine–indole hybrids belonging to series **41** (Figure 26) [122]. The spiropyrrolidine nucleus demonstrated significant inhibitory effects on both AChE and BChE, suggesting its potential as a crucial pharmacophore for the development of drugs targeting AD [25]. In the evaluation of the inhibitory activities of all compounds of series **41**, **41a** and **41b** (Figure 26) emerged as the most potent inhibitors of AChE (IC_50_ = 1.88 and 1.98 µM, respectively) and BChE (IC_50_ = 18.32 and 10.21 µM, respectively) displaying highest potency against the two ChE enzyme than the standard drug, galantamine. Molecular docking studies allowed to disclose the mode of binding and orientation into the active site of the enzymes: compounds **41a** and **41b** established a total of five and seven hydrogen bonds with hAChE, respectively, and engaged in interactions with PAS through hydrophobic interactions [122].

#### 4.1.4. Carbamate–Tryptamine Hybrids

Currently, many drugs used for AD treatment incorporate carbamate fragments in their structures [123] and recent advancements have been made in the development of carbamate-based inhibitors targeting ChE [124]. For instance, the drug molecule rivastigmine (Figure 1), which contains a carbamate group, has shown effectiveness in targeting both BChE and AChE in a pseudo irreversible manner, with a slight preference for BChE [125]. 

**Figure 27:**
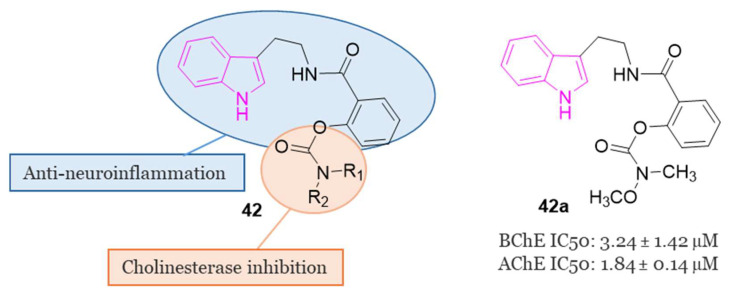
General chemical structure of carbamate-tryptamine hybrids of series **42**; chemical structure and biological activity of compound **42a**.

Given this widespread and crucial use of the carbamate structure in AD treatment, a series of innovative carbamate N-salicylate tryptamine derivatives of series **42** (Figure 27) were designed and synthesized in 2022 [75]. 

The design involved integrating pharmacophores based on N-salicyloyl tryptamine compounds with anti-inflammatory activity [126] and carbamate fragments with substantial impact in modulating ChE activity [124].

In the evaluation of AChE and BChE inhibitory activities, compound **42a** (Figure 27) demonstrated notable characteristics as a reversible dual inhibitor of both AChE and BChE, displaying IC_50_ values of 1.84 µM and 3.24 µM, respectively. The findings indicated that **42a** could reduce the levels of pro-inflammatory cytokines such as NO, iNOS, IL-6, TNF-α, and ROS, elevate the levels of anti-inflammatory cytokines like IL-4, and impede the aggregation of Aβ_1-42_. In a scopolamine-induced AD model in mice, behavioral tests revealed the efficacy of **42a** in enhancing learning and memory behaviors. Additionally, **42a** modulated the activities of cholinergic biomarkers in the hippocampus and prevented pathological changes in neurons within the hippocampal CA1 and CA3 regions. Notably, **42a** exhibited excellent pharmacokinetic properties and demonstrated permeability through the BBB [75]. 

**Figure 28:**
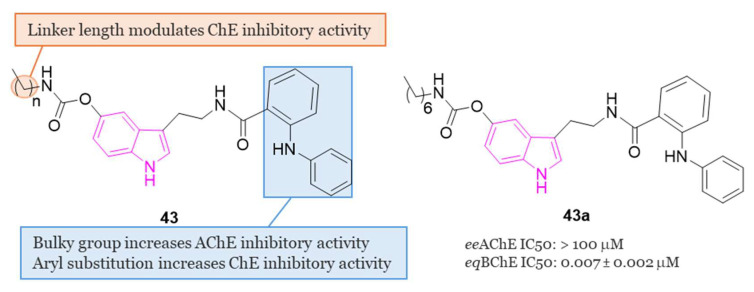
General chemical structure and SARs of carbamate N-anthraniloyl tryptamine hybrids of series **43**, chemical structure and biological activity of compound **43a**.

In this vein, in the same year, a series of N-anthraniloyl tryptamine derivatives **43** (Figure 28) as potential neuroprotective agents for the management of AD were synthesized and subjected to evaluation against eeAChE and eqBChE [127]. Results from the in vitro enzyme inhibition study revealed that compound **43a** exhibited the most potent inhibitory property against eqBChE, displaying IC_50_ value of 7 nM. Additionally, compound **43a** demonstrated robust antioxidant activity and showcased potential as a neuroprotective agent displaying also inhibitory effects on various inflammatory mediators, including IL-1β, IL-6, PGE2, TNF-α, IL-4, and IL-10. Safety profile of compound **43a** was confirmed through cell viability and toxicity studies. Most interestingly, in a mouse model of scopolamine-induced memory impairment, **43a** exhibited the ability to enhance learning and memory.

**Figure 29:**
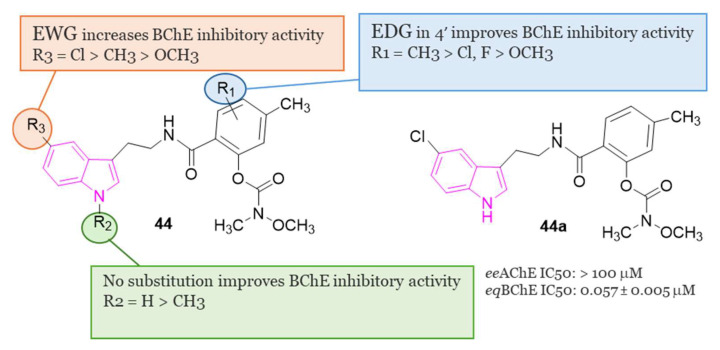
General chemical structure and SARs of carbamate N-salicyloyl tryptamine hybrids of series **44**, chemical structure and biological activity of compound **44a**.

In another study, Wang et al. [128] developed a series of N-salicyloyl tryptamine hybrids **44** (Figure 29), with compound **44a** emerging as the most potent inhibitor of BChE, displaying an impressive IC_50_ value of 0.057 μM in in vitro assessments. SAR analysis indicated that an electron-withdrawing group (EWG) on the tryptamine and an electron-donating group (EDG) on the phenyl group of the carbamate fragment enhance the ChE inhibitory property (Figure 29). Furthermore, **44a** demonstrated characteristics of a pseudo-irreversible inhibitor of BChE and displayed neuroprotective, antioxidant, and anti-neuroinflammatory properties. Finally, in vivo behavioral studies conducted on a scopolamine-induced mouse amnesia model revealed that compound **44a** significantly enhanced learning and memory.

#### 4.1.5. Tryptamine-Cinnamic Acid Hybrids 

**Figure 30:**
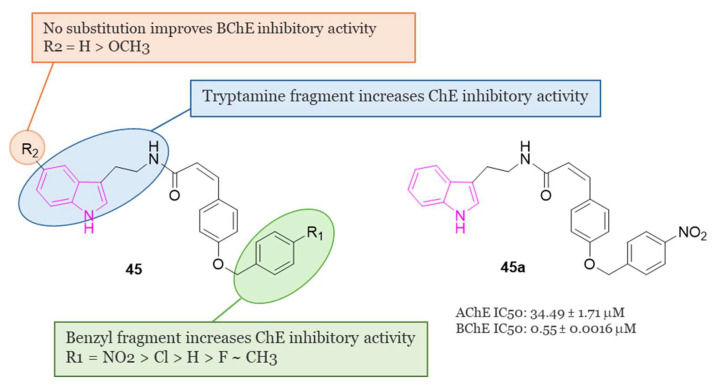
General chemical structure and SARs of tryptamine–cinnamic acid hybrids of series **45**; chemical structure and biological activity of compound **45a**.

Since cinnamic acid, a naturally occurring antioxidant, exhibits diverse biological activities associated with AD, such as free radical scavenging, metal chelation, and Aβ modulation, in 2018, Ghafary et al. [129] undertook the design and synthesis of a series of tryptamine-cinnamic acid hybrids. The cinnamic acid fragment was incorporated into tryptamine to confer multifunctional properties to the 12 compounds developed (series **45**, Figure 30), which were assessed in a study on AChE and BChE inhibition.

Results from enzyme inhibition studies revealed that most of the synthesized compounds demonstrated mild to moderate inhibitory activity against ChE, with IC_50_ values ranging from 14.18 to 39.1 µM against AChE and from 0.55 to 9.36 µM against BChE. Among these hybrids, **45a** (Figure 30) emerged as the most effective BChE inhibitor, displaying an IC_50_ value of 0.55 μM. Enzyme kinetic and molecular docking studies suggested that compound **45a** behaved as a mixed inhibitor for BChE binding both CAS and PAS.

#### 4.1.6. Tryptamine–Ferulic Acid Hybrids 

**Figure 31:**
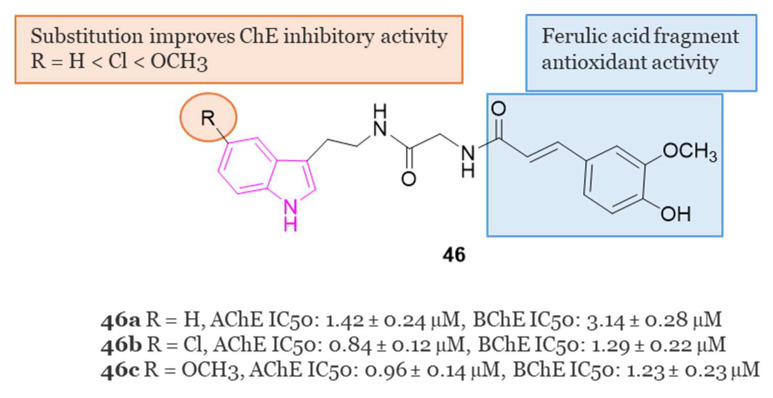
General chemical structure, SARs, and biological activities of tryptamine–ferulic acid hybrids **46a**–**c**.

In 2020, Singh et al. [130] synthesized a series of ChE inhibitors **46**, (Figure 31) inspired by natural sources for the treatment of AD: tryptamine and ferulic acid. The results of the biological assays indicated that all the developed compounds displayed preferential inhibition of hAChE over eqBChE. Notably, compounds **46a**–**c** (Figure 31) exhibited significant inhibition of hAChE and eqBChE with IC_50_ values of 1.42 µM, 0.84 µM, and 0.96 µM against AChE for **46a**, **46b** and **46c**, respectively. For BChE, the IC_50_ values were 3.14 µM, 1.29 µM, and 1.23 µM for compounds **46a, 46b** and **46c**, respectively.

Molecular modeling analysis indicated robust interactions between compound **46c** and key residues such as Trp86, Ser125, Glu202, Trp286, Phe295, and Tyr337 for AChE. Furthermore, compound **46c** exhibited mixed enzyme inhibition against both AChE and BChE, along with high antioxidant potency in the DPPH assay, displaying an IC_50_ value of 20.25 µM. It showed also notable metal chelation properties and the ability to mitigate H_2_O_2_-induced toxicity in SH-SY5Y cells.

In in vivo animal models, it was observed that a dosage of 10 mg/kg of **46c** served as a potent AChE inhibitor, enhancing learning and memory in a scopolamine-induced amnesia mouse model.

#### 4.1.7. Indole–Diosgenin Hybrids

**Figure 32:**
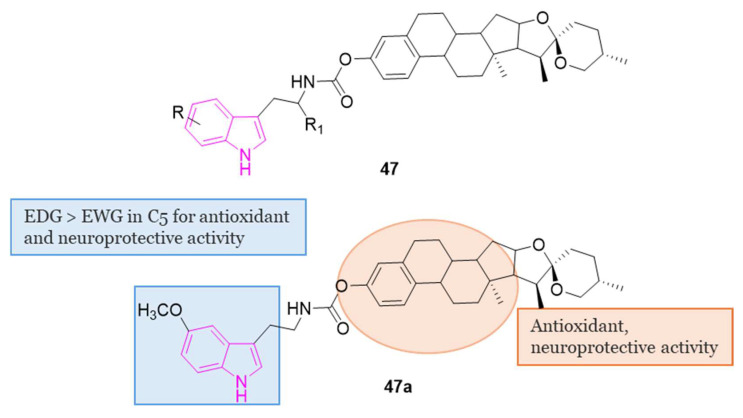
General chemical structure and SARs of indole–diosgenin hybrids of series **47**, chemical structure of compound **47a**.

Diosgenin belongs to the category of isospirostan steroids and has demonstrated neuroprotective properties by enhancing cognitive functions, reducing pro-inflammatory factors level, preventing Aβ-induced axonal atrophy, and addressing memory deficits [131]. Based on the neuroprotective effects of diosgenin and on the indole moiety, Zhou et al. (2021) synthesized 19 ester or carbamate-linked indole–diosgenin hybrids **47** (Figure 32) as potential dual-functional agents for AD treatment [132]. All hybrid molecules, except one, demonstrated no cytotoxicity to SH-SY5Y cells and exhibited superior neuroprotective activity compared to the parent compound diosgenin. Preliminary SAR highlighted that compounds featuring an EDG at C-5 of the indole ring displayed enhanced antioxidant activity compared to those with EWGs. Furthermore, carbamate-linked hybrids were found to be more potent than their ester-linked counterparts. Among the synthesized compounds, compound **47a** (Figure 32) emerged as the most promising neuroprotective agent. It demonstrated the ability to safeguard neuronal cells against H_2_O_2_- (52.9 ± 1.9%) and 6-hydroxydopamine (38.4 ± 2.4%)-induced oxidative stress, exhibited anti-Aβ toxicity (54.4 ± 2.7% against Aβ_1-42_), and improved cognitive functions in Aβ-damaged mice in the Morris water maze test. Additionally, the predicted values of brain/blood partition coefficient and polar surface area highlighted compound **47a** to have favorable absorption and permeation through the BBB. Finally, molecular docking studies indicated that compound **47a** binds strongly and with good affinity to Aβ_1-42_ by forming two hydrogen bonds with Lys28 and Glu22, potentially disrupting the formation of a salt bridge between these two amino acids, thereby inhibiting Aβ aggregation [132].

#### 4.1.8. Indolyl-Piperidine Hybrids

**Figure 33:**
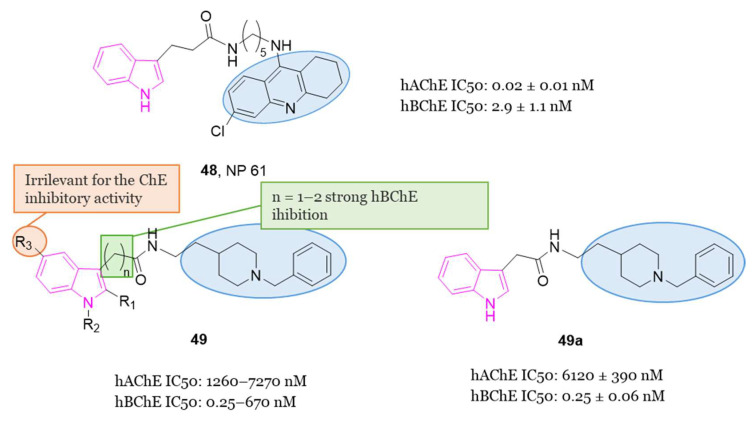
Chemical structure of compound **48** (NP61), general chemical structure, SARs, and biological activity of indolyl-piperidine hybrids of series **49**, chemical structure and biological activity of compound **49a**.

In the exploration of AChE dual binding site inhibitors, Garcia-Palomero et al. discovered the indole derivative **48** (NP61, Figure 33) exhibiting potent inhibition of AChE (activity in the subnanomolar range), effectively targeting both CAS and PAS simultaneously, and demonstrating the ability to hinder Aβ-amyloid aggregation in vitro (with an IC_50_ in the low micromolar range) [133]. 

Over a three-month period, oral administration of **48** effectively reversed cognitive impairment, as evidenced by improved performance in the Morris water maze test. Additionally, it led to a reduction in plaque accumulation within the brain of transgenic mice expressing human amyloid precursor protein [133]. 

Drawing inspiration from the dual ligands **48** (Figure 33) and donepezil (Figure 1), a further series (**49**, Figure 33) was developed in 2018. This series aimed to refine the structure of **48** while retaining the indole nucleus. The approach involved substituting the tacrine moiety (Figure 1) with the benzyl piperidine core present in donepezil. Additionally, variations were made in the chain length between the indole nucleus and the amide bridge, and different substituents were introduced at position 5 of the indole [134]. 

Interestingly, this series of indolylpiperidines **49** exhibited remarkable potency as selective inhibitors of BChE, with residual activity against AChE at low micromolar levels. Compound **49a** demonstrated the most potent hBChE inhibition, with an IC_50_ value in the subnanomolar range (0.25 nM), while inhibited hAChE in the micromolar range (6.12 μM).

SARs within this small library revealed that derivatives containing one or two methylene groups (n = 1 or 2) in the side chain exhibited strong hBChE inhibition in the nanomolar range, while the impact of the indole substituents on activity was found to be insignificant. The length of the chain appeared to be crucial for potency, highlighting the importance of one or two methylene groups in maintaining molecular flexibility.

#### 4.1.9. Miscellaneous Indole Hybrids

**Figure 34:**
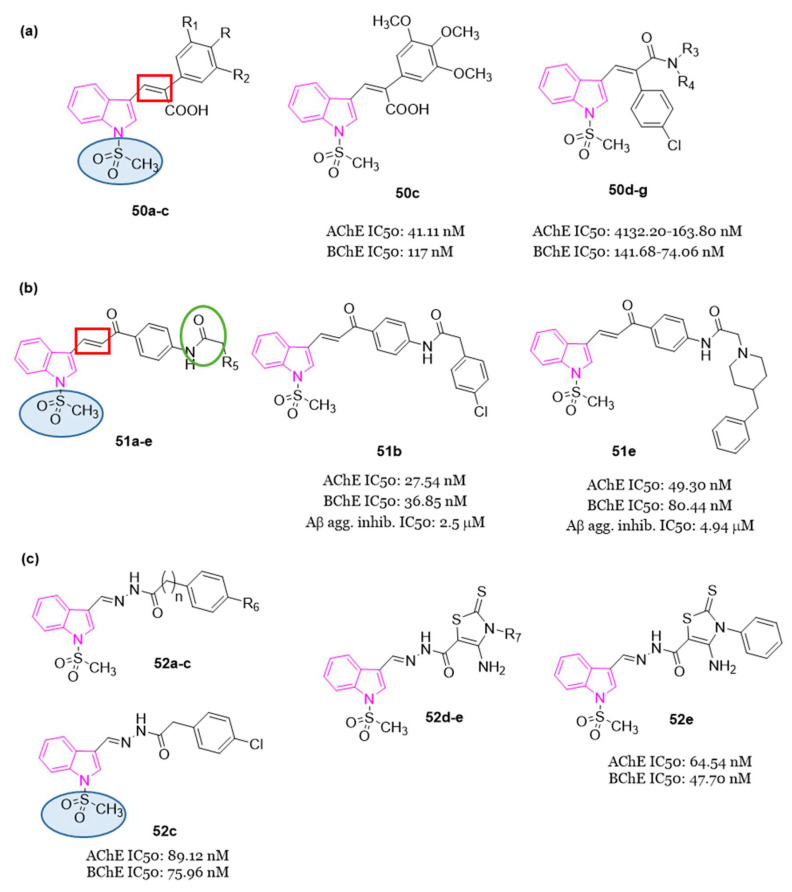
(**a**): General chemical structures of stilbene mimic derivatives (**50a**–**c** and **50d**–**g**), chemical structures and biological activities of compounds **50c** and **50d**–**g**; (**b**): General chemical structure of chalcone–donezepil-like derivatives (**51a**–**e**), chemical structures and biological activities of compounds **51b** and **51e**; (**c**): General chemical structures of hydrazone containing derivatives **52a**–**c** and **52d**–**e**, chemical structures and biological activities of compounds **52c** and **52e**.

In 2022, three series (**50**, **51** and **52**, Figure 34) of compounds featuring an indole nucleus as the primary framework, coupled with a methanesulfonyl group serving as a specific COX-2 pharmacophore, were conceptualized, synthesized, and assessed for their potential as remedies against AD and neuroinflammation [135]. 

Additionally, series **50** (Figure 34a) included a stilbene moiety like to resveratrol and ferulic acid, along with a piperazinyl pyrimidine motif and secondary amines. Series **50** and **51** incorporated a benzyl piperidine ring, reminiscent of donepezil. Series **51** (Figure 34b) introduced a chalcone segment and an NHCOCH_2_ linker present in certain derivatives utilized for anti-inflammatory purposes [136]. Meanwhile, series **52** (Figure 34c) featured a hydrazone moiety and a thiazole scaffold, as AD drugs integrating such moieties showed enhanced activity attributed to their ChE inhibitory, anti-Aβ aggregation, or anti-neuroinflammatory characteristics.

All compounds of series **50**–**52** underwent in vitro assessment for their inhibitory effects on AChE and BChE. The obtained results highlighted compound **50c** (Figure 34a), a stilbene carboxylic acid derivative, with superior AChE inhibitory activity (IC_50_ = 41.11 nM) compared to inhibition of BChE (IC_50_ = 117 nM). Conversely, stilbene amide derivatives **50d**–**g** (Figure 34a) displayed notable BChE inhibitory activity (IC_50_ = 41.68–74.06 nM) surpassing their AChE inhibitory effects (IC_50_ = 132.20–163.80 nM). Chalcone derivatives **51b** and **51e** (Figure 34b), along with hydrazone derivatives **52c** and **52e** (Figure 34c), demonstrated dual AChE/BChE inhibitory activities within the IC_50_ range of 27.54–89.12 nM and 36.85–80.44 nM, respectively. Compounds **51b** and **51e** emerged as the most potent inhibitors of self-induced Aβ-amyloid aggregation, with IC_50_ values of 2.50 and 4.94 μM, respectively, outperforming tacrine (IC_50_ = 3.5 μM). Evaluation of anti-inflammatory activity against various mediators (NO, COX-2, IL-1β, and TNF-α) revealed compounds **51b** and **51e** to possess a very good efficacy. Additionally, these compounds exhibited low toxicity towards neuroblastoma (SH-SY5Y) and normal hepatic (THLE2) cell lines. Subsequent drug-likeness and ADMET prediction analyses indicated that most tested compounds, including derivative **51b**, complied with Lipinski’s rule of five, suggesting their potential as promising candidates for further optimization towards the development of novel anti AD and anti-neuroinflammatory drugs [135].

**Figure 35:**
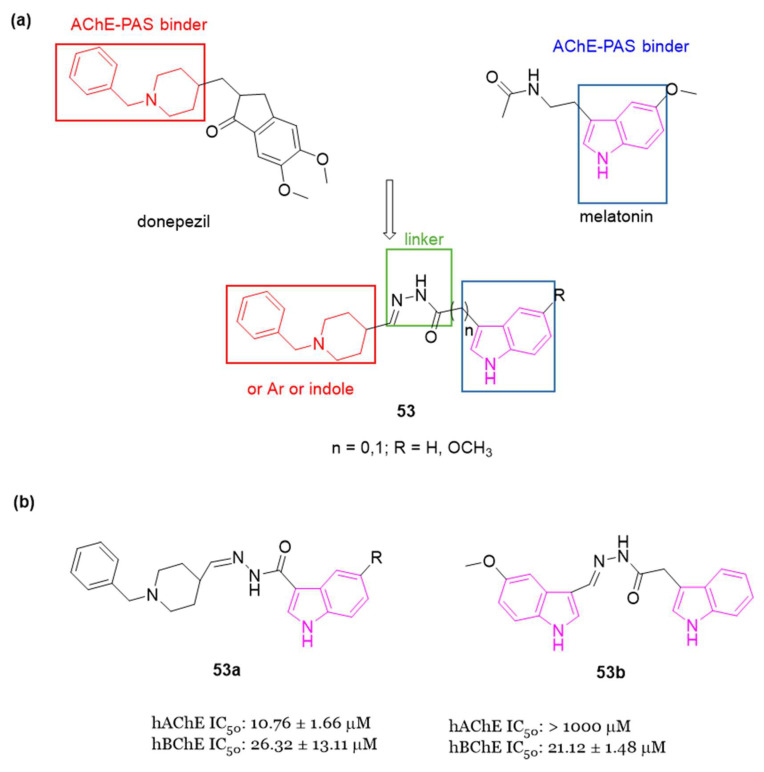
(**a**) Chemical structures of donepezil and melatonin and design of the new hybrids of series **53**; (**b**) Chemical structures and biological activities of compounds **53a** and **53b**.

In 2023 [137], a series of melatonin- and donepezil-based hybrid molecules bearing a hydrazone linker fragment (**53**, Figure 35) was designed, synthesized, and evaluated as MTDLs against AD-related neurodegenerative mechanisms. Initially, biological assays to determine the potential of the derivatives **53** for AChE/BChE inhibition were performed; then the in vitro antioxidant activities for the most promising molecules were investigated by DPPH and ABTS radical scavenging assays, Ferric Reducing Antioxidant Power (FRAP) methods and the inhibition of lipid peroxidation in the linoleic acid system by FTC methods. Among the indole derivatives, compound **53a** exhibited a well-balanced multifunctional profile, demonstrating interesting AChE inhibition (IC_50_ = 10.76 µM) and promising antioxidant activity. Compound **53b**, incorporating two indole moieties, showed the highest inhibitory activity (IC_50_ = 21.12 µM) and a high selectivity index hBChE / hAChE (47.34). Moreover, in vitro studies evinced that the melatonin derivative **53b** efficiently prevented H_2_O_2_-induced oxidative stress in SH-SY5Y cells. Compounds **53a** and **53b** showed low neurotoxicity (IC_50_ > 300 µM) against malignant neuroblastoma cell lines of human (SH-SY5Y) and murine (Neuro-2a) origin, as well as normal murine fibroblast cells (CCL-1), indicating their in vitro biocompatibility. In silico pharmacokinetics analysis predicted that all tested hybrids could be well absorbed, metabolized, and excreted. The experimental PAMPA-BBB study performed on the most interesting compounds revealed that compounds **53a** and **53b** were also capable of penetrating the BBB. The molecular docking studies indicated that compound **53a** could act as a ligand to both melatonin MT1 and MT2 receptors, as well as to AChE and BChE enzymes [137]. 

### 4.2. Indole Hybrids as BChE Inhibitors and 5-HT_6_R Antagonists 

#### 1-(Phenylsulfonyl)-1H-Indole Hybrids

**Figure 36:**
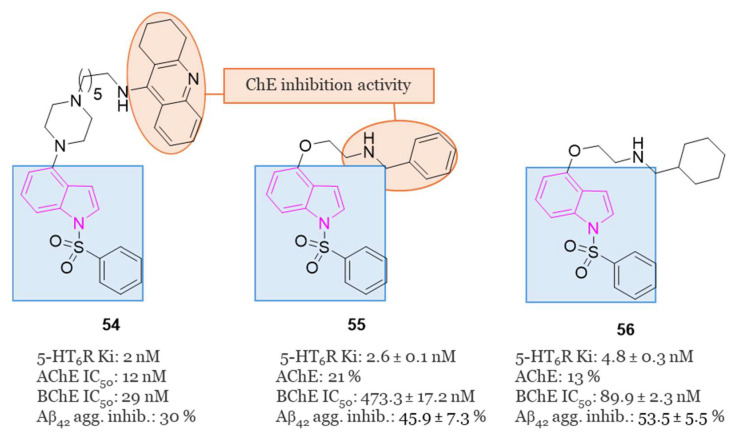
Chemical structures and biological activities of 1-(phenylsulfonyl)-1H-indole hybrids **54**–**56**.

In the development of MTDLs, various pharmacophore fragments were combined to provide compounds with significant in vitro ChE inhibitory activity, 5-HT_6_R antagonist potential, and amyloid β-anti-aggregation activity. In particular, recently [138,139], 1-(phenylsulfonyl)-4-(piperazin-1-yl)-1H-indole and tacrine resulted privileged structures providing a broad profile of biological activities, including in vitro inhibitory potency against tau and Aβ aggregation ranging from 30% to 61% at 10 μM, hBACE1 with IC_50_ values from 2 to 8 μM, 5-HT_6_R Ki values between 2 and 36 nM, hAChE IC_50_ values between 1 and 46 nM and eqBChE IC_50_ values in range from 8 to 21 nM. As one of the most potent, compound **54** (Figure 36) displayed well balanced potency against ChEs (AChE, IC_50_ = 12 nM; BChE, IC_50_ =29 nM), 5-HT_6_R (Ki = 2 nM), and anti-aggregating activity against Aβ (30% of inhibition at 10 μM) [138], so it can be regarded as a promising molecule for further development. However, it displays not-optimal physicochemical properties and poor drug-likeness because of its lipophilicity, flexibility and the too high aromatic ring count.

In this regard, in 2021, starting from compound **54**, fused MTDLs targeting BChE, 5-HT_6_ receptor and amyloid β aggregation with optimized drug-like properties were developed [76]. 

In particular, to optimize the physicochemical properties of compound **54**, the tacrine moiety was replaced with N-benzylamine, a donepezil-derived pharmacophore fragment, which was fused with 2-((1-(phenylsulfonyl)-1H-indol-4-yl)oxy)ethan-1-amine in order to obtain compound **55** (Figure 36), with favorable biological activity (5-HT_6_R, Ki = 2.6 nM; BChE, IC_50_ = 473 nM) and optimized physicochemical properties, complying with the Lipinski and Veber rules. 

Moreover, to explore and enhance the interactions of compound **55** with BChE, some modifications were made to the terminal benzylamine fragment. From the various synthesized compounds, derivative **56** (Figure 36) containing a cyclohexylmethanamine moiety in place of the benzylamine one, exhibited notable and well-balanced potencies against BChE (IC_50_ = 90 nM) and 5-HT_6_R (Ki = 4.8 nM), along with inhibitory activity against Aβ aggregation (53% at 10 μM). Additionally, it displayed favorable stability in mouse liver microsomes and a promising safety profile, prompting its selection for ADMET studies. Pharmacokinetic data from mouse experiments indicated that compound **56** effectively penetrates the BBB, with brain to plasma ratios of 3.33 and 6.79 following intravenous and oral administration, respectively. These findings highlighted compound **56** as a compelling candidate for in vivo pharmacology investigations [76]. 

**Figure 37:**
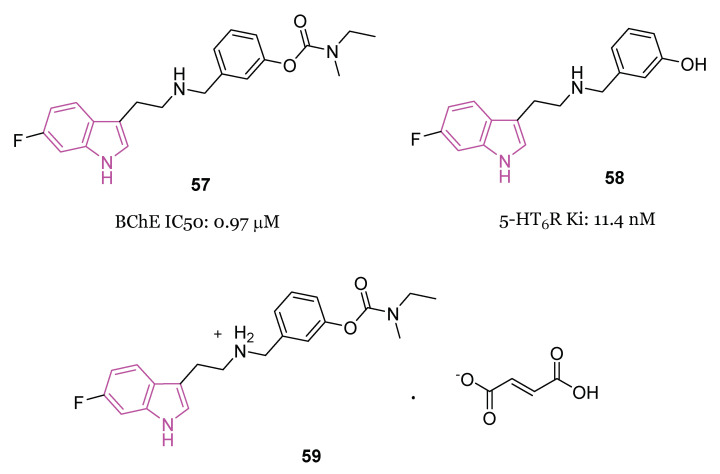
Chemical structures and biological activities of pleiotropic compound **57**, its metabolite **58** and its fumarate salt **59**.

In 2021 [77], a pleiotropic prodrug, **57** (Figure 37), was developed, whose design was inspired by the structural model of rivastigmine (Figure 1), a compound that covalently binds and inhibits both AChE and BChE. Subsequent studies revealed that compound **57** acts as a covalent inhibitor of BChE (IC_50_ = 0.97 μM). Upon hydrolysis, it releases compound **58** (Figure 37), an active metabolite associated with idalopirdine (Figure 13), that resulted a potent antagonist of 5-HT_6_ receptors (Ki = 11.4 nM). Furthermore, the fumarate salt of **57**, namely compound **59** (Figure 37), exhibited favorable druggability characteristics and demonstrated the ability to reverse memory deficits induced by scopolamine in vivo [77]. 

### 4.3. Indole Hybrids as MAO and 5-HT_6_R Inhibitors 

#### Indole-Piperazine Hybrids

**Figure 38:**
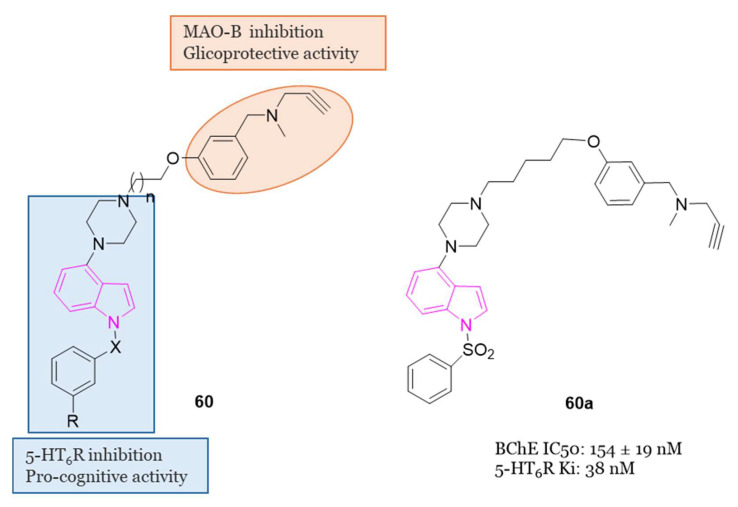
General chemical structure of indole–piperazine hybrids of series **60**, chemical structure and biological activity of compound **60a**.

In 2020, Canale et al. examined the impact of using pharmacophore hybridization strategy to obtain dual-acting 5-HT_6_R antagonists/MAO-B inhibitors. In particular, they linked a privileged scaffold of 5-HT_6_R, a (indol-4-yl)-piperazine fragment, with aryloxy fragments derived from MAO-B inhibitors, thus developing compounds of series **60** (Figure 38) [74].

Among the synthesized series, **60a** emerged as the most potent 5-HT_6_R/MAO-B dual inhibitor. Compound **60a** exhibited moderate metabolic stability in the rat microsomal assay and demonstrated favorable characteristics in terms of artificial membrane permeability. Moreover, it displayed no signs of hepatotoxicity and exhibited effective distribution to the brain. Additionally, in a model involving cultured astrocytes with 6-hydroxydopamine as the cytotoxic agent, compound **60a** demonstrated glioprotective properties. Lastly, in rats subjected to the novel object recognition (NOR) task and treated with scopolamine to induce memory deficits, **60a** completely reversed the cognitive impairments [74]. 

### 4.4. Indole Hybrids as ChE and MAO Inhibitors 

#### 4.4.1. Indole–Ladostigil–Carbamate/Urea Trihybrids

**Figure 39:**
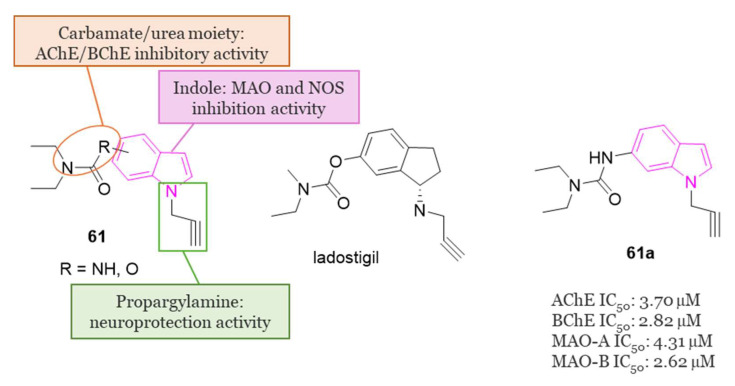
General chemical structure of derivatives of series **61**, chemical structure of ladostigil, chemical structure and biological activity of compound **61a**.

In the domain of pharmaceutical research, Joubert and his research team innovatively designed and synthesized a group of indole derivatives of general formula **61** (Figure 39), structurally inspired by the multimodal neuroprotective compound ladostigil (Figure 39), known for its ChE and brain-selective MAO inhibitory activities [140]. These compounds demonstrated the ability to inhibit hMAO-A, hMAO-B, eeAChE, and eqBChE, also providing neuroprotection [141]. Of particular note, compound **61a** showcased highly superior AChE and MAO-B inhibitory activities, outperforming ladostigil by factors of 9 and 14, respectively. It exhibited inhibitory activity against MAO-A, MAO-B, AChE, and BChE, with IC_50_ values of 4.31 µM, 2.62 µM, 3.70 µM, and 2.82 µM, respectively. Notably, compound **61a** displayed neuroprotective properties, with a substantial 52.62% protection against 1-methyl-4-phenylpyridinium (MPP+) induced damage to SH-SY5Y cells, when administered at a concentration of 1 µM.

#### 4.4.2. Indole–Donepezil–Chromone Trihybrids

**Figure 40:**
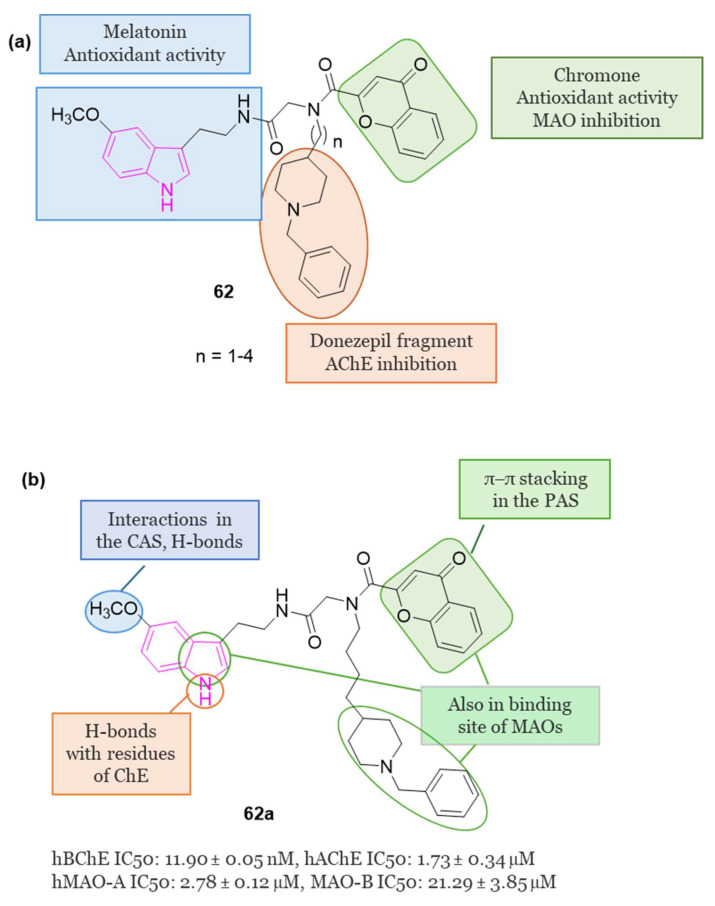
(**a**): General chemical structure of indole–donepezil–chromone trihybrids of series **62**; (**b**): Chemical structure, main interaction in the binding sites of ChEs and MAOs, and biological activity of compound **62a**.

In 2019, Pachon-Angona and colleagues conducted a synthesis of hybrid compounds within series **62** (Figure 40), specifically indole–donepezil–chromone hybrids (Figure 40). These compounds were designed with the aim of serving as antioxidants and inhibitors for hAChE/hBChE, as well as MAO-A/MAO-B [142]. 

Notably, from the library of 16 synthesized hybrids, compound **62a** (Figure 40) exhibited significant promise as a MTDL drug candidate. It demonstrated potent inhibition of hBChE (IC_50_ = 11.90 nM), hAChE (IC_50_ = 1.73 µM), hMAO-A (IC_50_ = 2.78 µM), and hMAO-B (IC_50_ = 21.29 µM). Additionally, this compound showcased robust antioxidant activity in vitro.

Upon molecular docking analysis, it was observed that **62a** occupied the entire enzymatic gorge of hAChE. Specifically, the chromone component interacted with Tyr124 and Trp286 through π–π stacking in the PAS, while the methoxy indole moiety formed interactions with Trp86 and Gly120 in the CAS binding pocket. Furthermore, the NH and OCH_3_ groups established two hydrogen bonds with Glu202 and Ser125 amino acid residues of CAS. Interestingly, the pyrrole ring of indole, N-benzyl piperidine, and chromone moieties were also found within the binding cavity of hMAO-A and -B.

#### 4.4.3. Indole–Miscellaneous Hybrids

**Figure 41:**
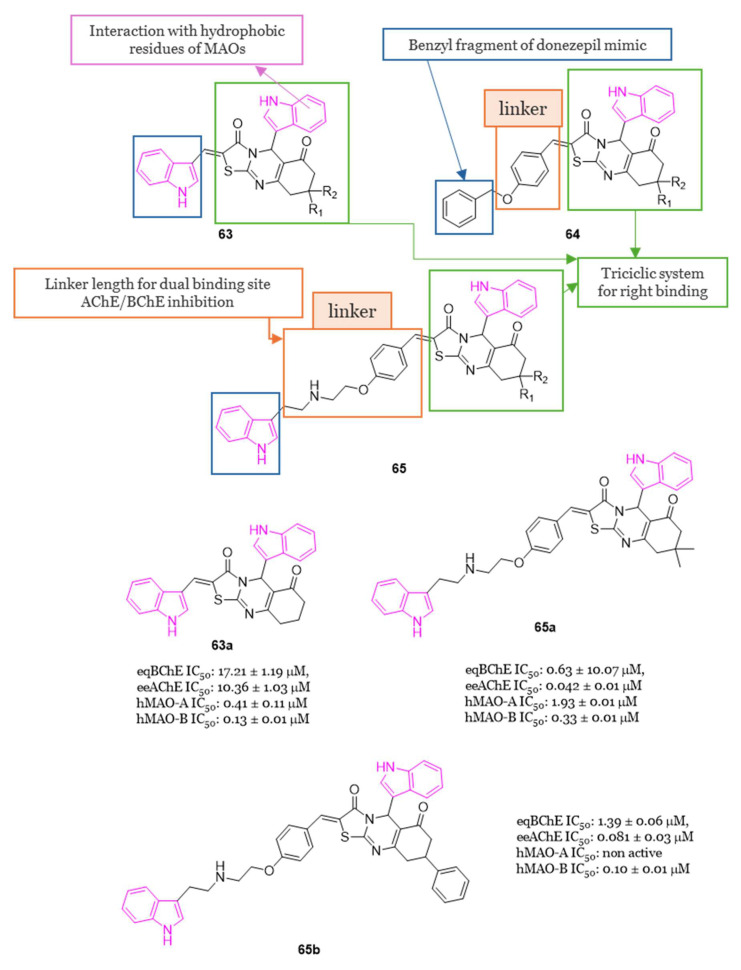
General chemical structure, main interactions in the binding sites of ChEs and MAOs of series **63**–**65** and chemical structures and biological activity of compounds **63a**, **65a** and **65b**.

In 2022 [143] some 2-arylidine derivatives of thiazolopyrimidine were synthesized and evaluated as multitarget inhibitors of ChEs and MAOs. In particular, three series (**63**–**65**, Figure 41) of compounds with different linker size and different target-anchoring functional groups were synthesized. 

Compounds of series **63** and **64** exhibited eeAChE inhibition in the range of micromolar to submicromolar concentrations, while compounds of series **65** showed very good ChE (both eeAChE and eqBChE) inhibition with IC_50_ values ranging from 42 nM to 1.39 μM. In particular compound **65a** was found to be a ChE inhibitor comparable to donezepil (Figure 1), taken as a reference drug (eeAChE IC_50_ = 0.05 μM and eqBChE IC_50_ = 5.4 μM). All compounds of the three series showed excellent MAO-B inhibition and selectivity relative to MAO-A with compounds **63a** and **65b** resulting the most potent MAO-B inhibitors of all the series **63**–**65**, with IC_50_ values of 0.13 μM and 0.10 μM, respectively. SAR studies evinced that the presence of a benzyloxy-benzylidene fragment (series **64**) enhanced the inhibition of ChEs compared with indolyl benzylidene moiety (series **63**). Studies performed to evaluate the acute toxicity of selected compounds, **63a** and **65a**,**b**, showed that the tested compounds are safe up to a 2000 mg/kg dose. The most active MAO-A/B inhibitor (**63a**) and the most active AChE and BChE inhibitor (**65a**) were selected for the PAMPA-BBB evaluation showing a good BBB permeability. The representative compounds **63a** and **65a**,**b** resulted non-neurotoxic at the tested concentrations (from 1 μM up to 40 μM in the MTT assay performed on neuroblastoma SHSY5Y cells). Finally, docking studies, carried out to correlate the experimental results, revealed that the binding pattern in the active site of AChE was characterized by interactions with the amino acid residues present in PAS and CAS sites via π−π stacking and hydrogen-bond interactions. Docking studies were also carried out on MAO isoforms evidencing that the binding orientation and interaction pattern correlated with the experimental results [143]. 

## 5. Conclusions and Future Perspectives

Neurodegenerative diseases (NDs) encompass a broad range of neurological disorders affecting millions worldwide, particularly prevalent among the elderly. Projections from the WHO suggest NDs will surpass cancer as the second leading cause of death within two decades, trailing only cardiovascular diseases. Urgent efforts to find effective treatments are warranted, as current drugs provide only temporary symptom relief without halting disease progression. ND involves the gradual loss of neurons in both the central and peripheral nervous systems, resulting in cognitive decline, brain deterioration, and impaired motor and behavioral functions. Alzheimer’s disease (AD) and Parkinson’s disease (PD) are the most common NDs, alongside others like Huntington’s disease (HD), amyotrophic lateral sclerosis (ALS), multiple sclerosis (MS), and various psychological disorders.

Common underlying causes of these diseases include oxidative stress, mitochondrial dysfunction, neuronal loss, inflammation, and abnormal protein aggregation. The etiology of NDs is complex, involving genetic, environmental, and lifestyle factors. Years of intensive research have uncovered genetic factors and shared biochemical pathways in various NDs, leading to the identification of eight distinctive hallmarks: pathological protein aggregation, synaptic and neuronal network dysfunction, proteostasis disturbances, cytoskeleton abnormalities, disrupted energy balance, DNA and RNA defects, inflammation, and neuronal cell death.

However, simplistic it may seem, with each ND characterized by specific neuronal loss or susceptibility and linked to a primary protein dysfunction, the reality is much more intricate. Our understanding of the causes of neuronal cell loss has evolved, acknowledging that the harmful effects of the various disease-associated proteins extend beyond a general toxicity of protein aggregates. Despite this, the precise mechanisms of toxicity for many implicated proteins remain elusive. This presents a challenge, as incomplete comprehension hinders both the development of therapeutic strategies and the anticipation of potential effects beyond disease modification. Yet, there’s growing recognition that NDs often exhibit multiple pathologies, with overlapping pathways and targets. This suggests that future treatments may be guided by tissue pathology and genetic factors rather than clinical phenotype. Although addressing underlying pathophysiology holds promise for treating NDs, it remains in its infancy, with significant hurdles to overcome, particularly the imperative to intervene early in the disease progression. Achieving this will necessitate a reevaluation of diagnostic criteria and the development of tools for earlier detection [144]. 

In line with this perspective, innovative therapeutic approaches for neurodegenerative conditions are under exploration. One promising strategy involves leveraging the regenerative potential of mesenchymal stem cell (MSC) transplantation. MSCs have exhibited the capacity to modulate the immune response, stimulate the growth of nerve fibers, induce blood vessel formation, and facilitate tissue repair, likely through the secretion of extracellular vesicles (EVs). These EVs derived from MSCs appear to retain some of the beneficial properties of their parent cells, such as the ability to regulate nerve fiber growth, promote blood vessel formation, and aid in tissue healing. Consequently, it is reasonable to speculate that deciphering the mechanisms by which MSC-derived EVs exert their therapeutic effects could pave the way for the development of novel therapeutic interventions in the realm of NDs in the near future [145].

Anyhow, it is certain that to date in the treatment of NDs small compounds still maintain an advantage due to their simple structures, facilitating their entry into the CNS, and being more cost-effective compared to larger biological substances, rendering them more appealing to patients. Furthermore, the identification of highly targeted small molecules with superior BBB permeability, crucial for innovative NDs treatment, can be facilitated by computational methods like virtual screening (VS). This approach rapidly provides a pool of biologically active compounds in a cost-effective manner. Recently, the integration of artificial intelligence techniques such as deep and machine learning has enhanced small molecule drug discovery, increasing success rates by addressing challenges inherent in traditional ligand- and receptor-based VS methods. Moreover, since artificial intelligence (AI) and machine learning (ML) represent highly effective tools for integrating various types of data, including transcriptomic, structural, and clinical data, they have a great potential for repurposing drugs to treat NDs [146,147]. Drug repositioning, also known as drug repurposing, involves identifying new uses for existing drugs that have been approved for other indications or have been declined/abandoned in development. This strategy breathes new life into established drugs by repurposing them for novel therapeutic applications. Several studies have showcased the success of drug repurposing such as, sildenafil, thalidomide, and aspirin. Leveraging drug repositioning offers a pertinent and cost-effective approach to discovering new medicinal opportunities. For instance, kinase inhibitors originally developed for oncology purposes have shown significant neuroprotective effects in NDs. Given its potential to save time and resources, drug repositioning may emerge as a crucial method for uncovering new therapeutic possibilities for existing drugs or drug candidates, particularly in the realm of NDs [148].

Therefore, while much remains to be understood about the mechanisms and biomarkers of NDs, significant research and clinical development efforts are necessary to optimize existing molecules and mitigate potential side effects. Certainly more in vivo experiments are needed and, due to the complexity of causes underlying NDs, it seems plausible to suggest that combined approaches might allow for more effective therapies. Moreover, it appears imperative that scientific, clinical and pharmaceutical efforts continue to pursue strategies devoted to the improvement of drug delivery with chemical modifications, bioconjugations and nanocarriers. The ultimate goal must be to significantly delay, if not prevent, disease progression and early death in patients with NDs.

It’s well-documented that heterocyclic compounds play a crucial role in drug development, offering diverse pharmacotherapeutic properties. Among these, nitrogen-containing heterocycles have garnered significant attention from regulatory bodies like the FDA due to their ability to engage hydrogen bonds or polar interactions, facilitating their effective binding to molecular targets and their subsequent pharmacological effects. Indole, a naturally occurring heterocyclic aromatic ring system, has emerged as a key player. Comprising a fused six-membered benzene ring and a five-membered pyrrole ring, its electronic and steric properties make it conducive to achieve both bioavailability and pharmacological efficacy. Recognized as a versatile pharmacophore, indole interacts with a multitude of receptors, leading to a broad spectrum of biological activities. Furthermore, by expanding the click chemistry concept wherein modular synthesis is used for rapid functional discovery, recently an indole based on-plate parallel synthesis has been developed as a powerful tool for rapid library construction and lead discovery [149]. This reaction is modular, robust and highly site-selective, employs a simple and mild reaction system, leading to high yields with excellent functional group compatibility, thus enabling to easily enrich the chemical space of indole medicinal chemistry. For all these reasons, indole certainly stands as one of the most privileged structure in medicinal chemistry, serving as a core framework in numerous therapeutic agents with diverse activities, including anti-neurodegenerative properties.

In this context, the present review summarized the rational design strategies employed by various research teams in the development of anti-neurodegenerative indole-based compounds which have the potential to modulate various molecular targets, with reference to the most recent advances between 2018 and 2023.

In addressing NDs, recent research has been focused on various molecular targets and the indole-based derivatives discussed herein primarily interact with ChE, β-amyloid, MAO-B, A_2A_AR, PERK signaling, AMPK, and 5-HT_6_R. Furthermore, recognizing the complex nature of ND pathophysiology, adopting a multifunctional approach by individual molecules proves advantageous in advancing new drug candidates. In this view, also the incorporation of the indole moiety with diverse bioactive components has been employed in the development of indole-based hybrids as MTDLs useful in NDs. 

Most of the single-targeted compounds or MTDLs developed demonstrated to be effective in moderating inhibition of ChEs, hinder Aβ aggregation, and exerting antioxidant or anti-neuroinflammatory properties. SAR investigation furnished comprehensive insights into the design and rationale of the synthesized compounds.

In most of the studies performed, in vitro studies on human neuroblastoma cell lines, mainly SH-SY5Y, were involved, and some of the indole-based compounds reported were found to have promising activity to enter the preclinical studies phase.

As a summary, the properties of the most interesting compounds reviewed have been schematized in Table 2. 

Through this review, the objective was to offer insights into the current data regarding indole-based derivatives in the treatment of NDs, with the intention of assisting medicinal chemists in formulating and advancing the creation of enhanced and highly targeted novel compounds for both in vitro and in vivo investigations. This effort aimed to facilitate the discovery and introduction of more potent drug candidates for the treatment of NDs, with improved efficacy and enhanced clinical utility.

## Figures and Tables

**Table 1:** Biological activities of compounds **30a**, **30b** and **31a**, **31b**.

Comp.	Experimental ChE Inhibitory Activity		IC_50_ Values (μM) DPPH Free Radical	A_0.s_ Values (μM)	
	hAChE (μM)	hBChE (μM)		ABTS Cation Radical	CUPRAC
**30a**	30.39 ± 0.24	7.42 ± 0.04	73.73 ± 0.43	17.38 ± 0.29	19.88 ± 1.10
**30b**	59.37 ± 0.38	1.95 ± 0.02	45.37 ± 0.46	17.66 ± 0.49	21.64 ± 0.92
**31a**	74.46 ± 0.45	42.01 ± 0.27	26.88 ± 1.03	85.19 ± 1.41	17.84 ± 1.07
**31b**	66.79 ± 0.91	8.48 ± 0.09	27.65 ± 0.85	69.33 ± 1.29	16.28 ± 0.80
**Galantamine**	19.86 ± 0.17	40.90 ± 0.12	-	-	-
**BHA**	-	-	45.95 ± 0.32	17.59 ± 0.10	18.44 ± 0.15
**BHT**	-	-	58.86 ± 0.50	13.25 ± 0.27	26.64 ± 0.14
**α-tocopherol**	-	-	16.30 ± 0.79	9.74 ± 0.42	-

**Table 2:** Properties of the most interesting compounds reviewed.

Cpd	Class	Target	Activity In Vitro	Activity In Vivo	Other Characteristics	Possible Use in ND	Ref.
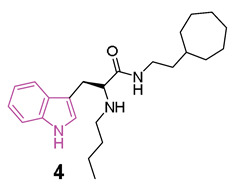	Tryptophan analogue ChE inhibitor	hBChE	hBChE IC_50_: 22 nM	Improvement in long-term memory and spatial long-term memory retrieval in AD mouse model	High permeability across the BBB	Alzheimer disease	[36]
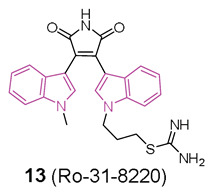	Indole-based protein aggregate inhibitor	PKCα	In SH-SY5Y cell line reduced PKCα activity and the tau phosphorylation	Reversed tau-induced memory impairment, and improved midge motor functions	-	Frontotemporal dementia	[48]
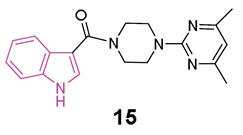	Indole-piperazine-pyrimidine A_2A_AR antagonist	A_2A_AR	Affinity human A_2A_AR Ki = 8.7 μM	In the Drosophila model of PD enhanced movement and mitigated the loss of dopaminergic neurons	-	Parkinson disease	[60]
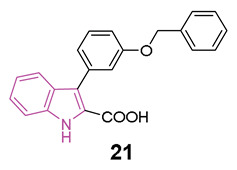	Indole-based AMPK activator	AMPK	AMPK activation in HEK293 cell lines	Effectiveness in animal models for HD	Favorable in silico druggability profile	Hangtinton disease	[65]
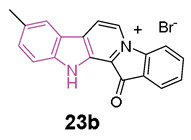	Multifunctional fascaplysin derivative	ChEs	AChE IC_50_: 0.95 µM BChE IC_50_: 2.79 µM reduced neurotoxicity in the nanomolar range	Improve cognitive impairment in mice, without impacting locomotor functions	Capability to penetrate the BBB, lower in vivo acute toxicity than fascaplysin	Alzheimer disease	[88]
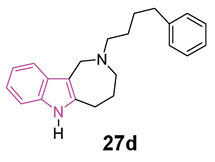	Multifunctional 1,2,3,4,5,6-hexahydroazepino[4,3-b]indole derivative	ChEs Aβ agg.	eeAChE IC_50_: 20.00 µM hsBChE IC_50_: 0.17 µM significant protective effects	Not carried out yet	-	Alzheimer disease	[92]
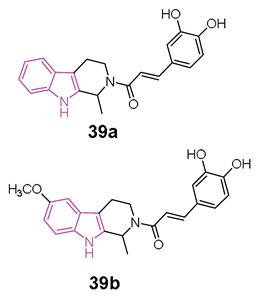	Multifunctional cinnamic acid- β-carbolines hybrids	ChEs Aβ agg.	**39a** BChE IC_50_: 1.32 μM **39b** BChE IC_50_: 6.47 μM non-neurotoxic effects, in PC12, SHSY5Y, BV-2, HT22, and L02 cell lines. reduced ROS production in BV2 cells	In AD mice model, orally administered **39a** and **39b** restored learning and memory function	-	Alzheimer disease	[119]
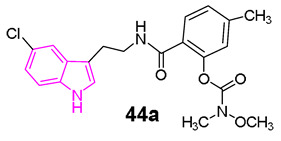	Multifunctional carbamate- tryptamine hybrid	ChEs	BChE IC_50_: 0.057 μM neuroprotective, antioxidative, anti-neuroinflammatory	Significantly enhanced learning and memory in a scopolamine-induced mouse amnesia model	-	Alzheimer disease	[128]
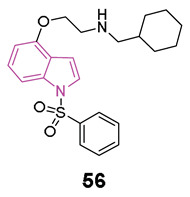	Multifunctional 1-(phenylsulfonyl) -1H-indole hybrid	BChE 5-HT_6_R Aβ agg.	BChE IC_50_: 89.9 nM 5-HT_6_R Ki: 4.8 nM Aβ_42_ agg. inhib.: 53.5 %	Not carried out yet	Effectively penetrates the BBB		[76]
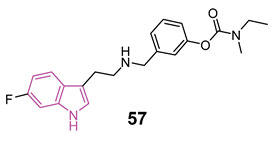	Multifunctional carbamate -tryptamine hybrid	BChE 5-HT_6_R	BChE IC_50_: 0.97 μM. 5-HT_6_R Ki: 11.4 nM	Ability to reverse memory deficits induced by scopolamine in vivo	Favorable druggability characteristics	Alzheimer disease	[77]
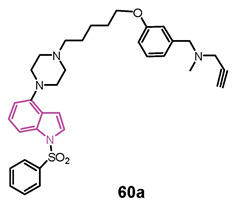	Multifunctional indole-piperazine hybrid	5-HT_6_R MAO-B	BChE IC_50_: 154 nM 5-HT_6_R Ki: 38 nM	Completely reversed the cognitive impairments in rats subjected NOR task and induced with scopolamine to memory deficits	Metabolic stability, artificial membrane permeability, no hepatotoxicity, good brain distribution	Alzheimer disease	[74]

## List of Abbreviations

Aβ: amyloid-β; A_2A_AR: A_2A_ adenosine receptor; ABTS: 2,2′-azino-bis(3-ethylbenzothiazoline-6-sulfonic acid; Ach: acetylcholine; AChE: acetylcholinesterase; AD: Alzheimer disease; ALP: autophagy-lysosome pathway; ALS: amyotrophic lateral sclerosis; AMP: adenosine monophosphate; AMPK: adenosine monophosphate-activated protein kinase; APOE: apolipoprotein E; APP: amyloid precursor protein; APP Aβ precursor protein; BACE-1: beta-site APP cleaving enzyme 1; α-Syn: α-synuclein; BBB: blood–brain barrier; BChE: butyrylcholinesterase; BHA: butylated hydroxyanisole; BHT butylated hydroxytoluene; C9ORF72: chromosome 9 open reading frame 72; CAS: catalytic anionic sites of acetylcholinesterase; cdk5: cyclin-dependent kinase-5; CHO: Chinese hamster ovary; CNS: central nervous system; D_2_R: dopamine D_2_ receptor; DPPH: 2,2-diphenyl-1-picrylhydrazyl; EAE: experimental autoimmune encephalomyelitis; ECS: endocannabinoid system; EMA: European Medicines Agency; ER: endoplasmic reticulum; FAAH: fatty acid amide hydrolase; FDA: Food and Drug Administration; FRAP: ferric reducing antioxidant power; GPCR: G protein-coupled receptor; GSK3:glycogen synthase kinase 3; HD: Huntington disease; IgG1: immunoglobulin G1; LPS: lipopolysaccharide; MAO-A monoamine oxidase A; MAO-B monoamine oxidase B; MAPs: microtubule-associated proteins; MPP+: 1-methyl-4-phenylpyridinium; MPTP: 1-methyl-4-phenyl-1,2,3,6-tetrahydropyridine; MS: multiple sclerosis; MTDLs: multitarget-directed ligands; NDs: Neurodegenerative diseases; NF-κB: nuclear factor κB; NFTs: neurofibrillary tangles; NMDA: N-methyl-D-aspartate; NOR: novel object recognition; NTRK1: neurotrophic receptor tyrosine kinase 1; PAMPA: parallel artificial membrane permeability assay; PAS: peripheral anionic sites of acetylcholinesterase; PD: Parkinson disease; PERK: protein kinase R-like endoplasmic reticulum kinase; PHFs: paired helical filaments; P-gp: P-glycoprotein; PIGAs: phenylindolylglyoxylamides; PKCa: protein kinase C alpha; PPMS: primary progressive multiple sclerosis; PS1: presenilin 1; P-tau: hyperphosphorylated tau; ROS: reactive oxygen species; ROT: rotenone; RRMS: relapsing-remitting multiple sclerosis; SARs: structure–activity relationships; 5-HTR_6_: serotonin type 6 receptor; SFs: straight filaments; SIRT1: sirtuin 1; SNpc: substantia nigra pars compacta; SOD1: superoxide dismutase 1; SPs: senile plaques; TDP-43: TAR DNA binding protein 43; TH: tyrosine hydroxylase; WHO: World Health Organization.
